# The Dynamics of Adaptation to Stress from Standing Genetic Variation and de novo Mutations

**DOI:** 10.1093/molbev/msac242

**Published:** 2022-11-05

**Authors:** Sandra Lorena Ament-Velásquez, Ciaran Gilchrist, Alexandre Rêgo, Devin P Bendixsen, Claire Brice, Julie Michelle Grosse-Sommer, Nima Rafati, Rike Stelkens

**Affiliations:** Division of Population Genetics, Department of Zoology, Stockholm University, Svante Arrheniusväg 18 B, 106 91 Stockholm, Sweden; Division of Population Genetics, Department of Zoology, Stockholm University, Svante Arrheniusväg 18 B, 106 91 Stockholm, Sweden; Division of Population Genetics, Department of Zoology, Stockholm University, Svante Arrheniusväg 18 B, 106 91 Stockholm, Sweden; Division of Population Genetics, Department of Zoology, Stockholm University, Svante Arrheniusväg 18 B, 106 91 Stockholm, Sweden; Division of Population Genetics, Department of Zoology, Stockholm University, Svante Arrheniusväg 18 B, 106 91 Stockholm, Sweden; Division of Population Genetics, Department of Zoology, Stockholm University, Svante Arrheniusväg 18 B, 106 91 Stockholm, Sweden; Faculty of Science and Engineering, Maastricht University, Paul-Henri Spaaklaan 1, 6229 EN Maastricht, The Netherlands; Department of Medical Biochemistry and Microbiology, National Bioinformatics Infrastructure Sweden, Uppsala University, SciLifeLab, 751 23 Uppsala, Sweden; Division of Population Genetics, Department of Zoology, Stockholm University, Svante Arrheniusväg 18 B, 106 91 Stockholm, Sweden

**Keywords:** microbial experimental evolution, yeast, adaptation dynamics, environmental stress, parallelism, time series, Pool-Seq

## Abstract

Adaptation from standing genetic variation is an important process underlying evolution in natural populations, but we rarely get the opportunity to observe the dynamics of fitness and genomic changes in real time. Here, we used experimental evolution and Pool-Seq to track the phenotypic and genomic changes of genetically diverse asexual populations of the yeast *Saccharomyces cerevisiae* in four environments with different fitness costs. We found that populations rapidly and in parallel increased in fitness in stressful environments. In contrast, allele frequencies showed a range of trajectories, with some populations fixing all their ancestral variation in <30 generations and others maintaining diversity across hundreds of generations. We detected parallelism at the genomic level (involving genes, pathways, and aneuploidies) within and between environments, with idiosyncratic changes recurring in the environments with higher stress. In particular, we observed a tendency of becoming haploid-like in one environment, whereas the populations of another environment showed low overall parallelism driven by standing genetic variation despite high selective pressure. This work highlights the interplay between standing genetic variation and the influx of de novo mutations in populations adapting to a range of selective pressures with different underlying trait architectures, advancing our understanding of the constraints and drivers of adaptation.

## Introduction

Ongoing climate change and sudden anthropogenic forces drive species outside of their ecological comfort zone (Radchuk et al. 2019). To better manage at-risk populations, a deeper understanding of adaptation dynamics from different sources of variation and under different types of selection pressures is required (Matuszewski et al. 2015; Bitter et al. 2019; Lai et al. 2019; Chaturvedi et al. 2021). Experimental evolution with microbial systems paired with time-series whole-genome sequencing is a powerful tool to study adaptation and the underlying genetic mechanisms leading to fitness change. Many experiments start from clonal populations, that is, initially isogenic lineages, adapting and diverging over time by accumulating new mutations (e.g., Lenski et al. 1991; Good et al. 2017; Johnson et al. 2021). This allows for the modelling of adaptation dynamics by selection on de novo mutations, as is the case in highly homogenous populations such as clonal pathogens in plant monocultures upon pesticide treatment or seasonal clonal turnover in *Daphnia* (McDonald et al. 2016; Mcdonald and Stukenbrok, 2016). However, this demographic scenario does not reflect the genetic starting conditions of more heterogeneous natural populations facing rapid environmental change. Adaptation dynamics from standing genetic variation differ substantially from the dynamics of isogenic populations. For instance, adaptation from standing genetic variation is more likely to lead to the fixation of more alleles of small effect relative to de novo mutations, imposing constraints on allele frequency dynamics through linkage, epistasis, and pleiotropy (Barrett and Schluter 2008; Thompson et al. 2019). In sexual populations, adaptation from standing genetic variation can involve partial selective sweeps and subtle shifts of allele frequencies, whereas single de novo mutations are associated with hard sweeps (Barghi et al. 2020). But in asexual populations, clonal interference is a primary factor determining patterns of allele frequency change (Gerrish and Lenski 1998; Lang et al. 2013), which is often only discussed in terms of de novo mutations.

Recently, several studies with yeast have explored the role of standing genetic variation in diverse contexts, such as evolution under different levels of outcrossing and ploidy (Kosheleva and Desai 2018), the dissection of traits’ genetic architecture (Parts et al. 2011; Cubillos et al. 2013; Linder et al. 2020), as well as levels of genetic parallelism among replicated populations (Burke et al. 2014). These studies found ample evidence of polygenic adaptation involving loci spread all across the genome (Burke et al. 2014; Kosheleva and Desai 2018), with outcrossing improving the power for genetic mapping (Parts et al. 2011; Cubillos et al. 2013). Usually the same alleles increase in frequency across replicates, as expected from large populations adapting from the same genetic diversity (MacPherson and Nuismer 2017). However, the total number of treatments is often small in long-term microbial experimental evolution studies, where only one or two different environments, often just laboratory adaptation along with one stressor, are tested (but see Bailey et al. 2017). Theory predicts that stronger selection should lead to higher levels of parallelism from both de novo mutations (Orr 2005) and standing genetic variation (MacPherson and Nuismer 2017), but differences in experimental design and methodology limit comparisons across studies. Thus, multiple environments within the same experiment are required to investigate this prediction (Bailey et al. 2015).

In addition, there is mixed evidence regarding the role of de novo mutations in the presence of standing genetic variation during asexual reproduction. On relatively short-time scales (<100 generations), de novo mutations are expected to be too rare to have an impact, as they must have very large effects in order to reach detectable frequencies (Illingworth et al. 2012). But as the time-scale increases, de novo mutations of smaller effects have more opportunity to increase in frequency. Most short- to medium-term microbial evolution studies (∼100–600 asexual generations) agree that de novo mutations have little impact on the evolution of outbred experimental populations (Illingworth et al. 2012; Burke et al. 2014; Phillips et al. 2020; Wing et al. 2020). A long-term study reached similar conclusions even after ∼960 asexual generations (Kosheleva and Desai 2018). In contrast, another study found dozens of de novo mutations associated with high fitness after only ∼54 asexual generations in a similarly outbred population (Vázquez-García et al. 2017). Note however that these mutations occurred during 12 rounds of sexual reproduction prior to experimental selection. Intuitively, standing genetic variation in an asexual population should be eroded by the eventual fixation of a single genotype, but is this process so fast that beneficial de novo mutations have no effect on the fate of ancestral variation?

**Fig. 1.**
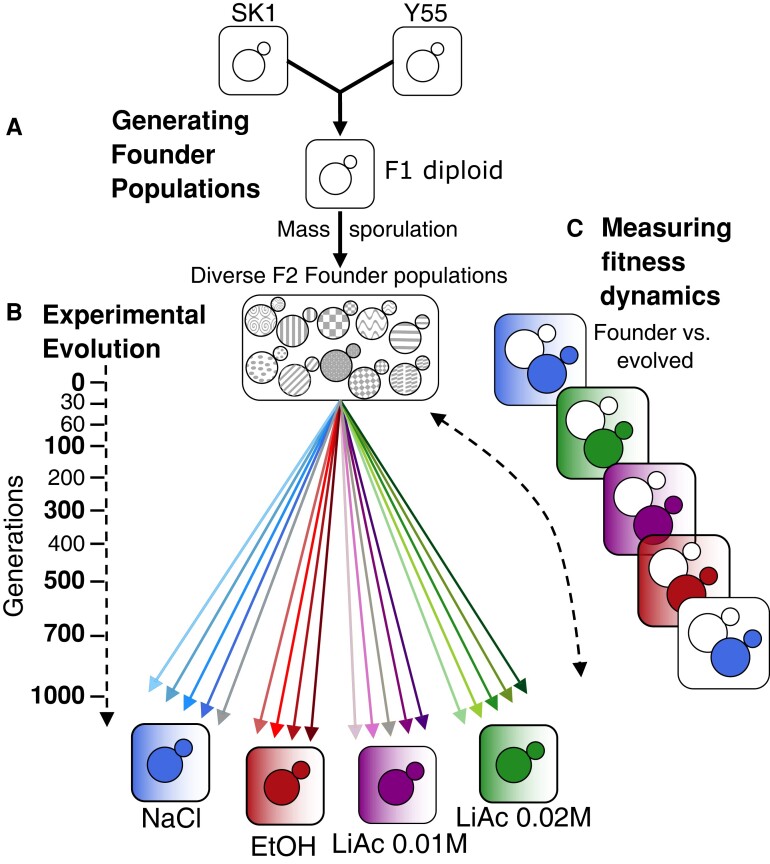
Experimental design. (*A*) Diverse founder populations were generated by mass sporulation of a cross between strains Y55 and SK1. (*B*) Asexual yeast populations were evolved for 1,000 generations in four environments: NaCl 0.75 M, EtOH 8%, LiAc 0.01 M, and LiAc 0.02 M. Shaded squares indicate selective media; white squares are the ancestral medium (SC). Replicate populations are indicated as separate arrows. Sampling time points for sequencing are indicated on the dotted arrow. One population of NaCl and LiAc 0.01 M, as well as the generation 1,000 of both LiAc treatments and of two populations of NaCl, were lost due to contamination. (*C*) Fitness assays using OD_600_ after 24 h of growth were used to compare founders and evolved populations at six time points of evolution in selective and ancestral environments. Only a subset of all pairwise comparisons is shown. Assays were carried out at six time points (marked in bold on the left).

Here, we address the impact of genetic and environmental diversity on adaptation dynamics, by using experimental microbial evolution with genetically diverse *Saccharomyces cerevisiae* yeast populations in four environments, each with different fitness costs. Founder populations were generated by crossing two different strains of yeast (Y55 and SK1), in a similar spirit to that of Kosheleva and Desai (2018). The F1 offspring was mass-sporulated to generate a diverse founder population of recombined, genetically unique F2 genotypes. Replicate populations of these founders thus contained millions of different haplotypes (fig. 1) before we subjected them to selection in different environments for up to 1,000 generations. The fitness of evolved populations was compared with the fitness of the founder population at five time points of evolution (in ancestral and stressful environments), and populations were pooled and sequenced at nine time points to track changes in genetic variation over time. We explored the dynamics of ancestral and de novo allele frequencies, as well as the variation in chromosome copy numbers, as aneuploidies can play an important role in adaptation of yeast to stressful environments (reviewed in Gilchrist and Stelkens 2019). Our design allowed us to assess the degree of parallel molecular and phenotypic evolution in replicate populations selected in the same and different environments. We made two broad predictions: (1) the degree of parallelism between replicates is larger in more stressful environments, and (2) adaptation will be driven by standing genetic variation first and by de novo mutations later in the experiment.

## Results

### Fitness Dynamics

**Fig. 2.**
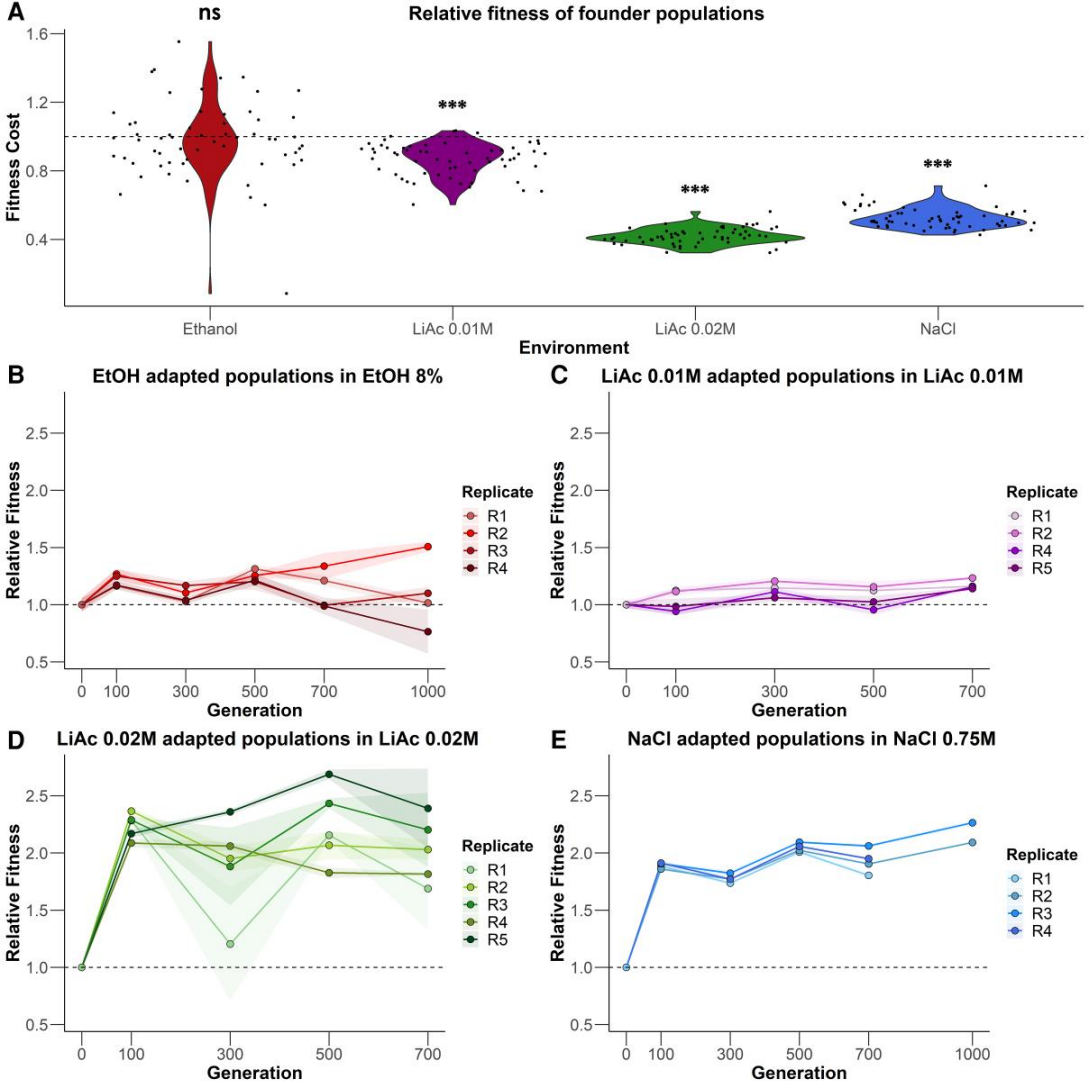
Fitness dynamics. (*A*) Founder population fitness (OD_600_ after 24 h of growth) in selective environments compared with growth in ancestral conditions (SC medium). Values of 1 (dashed horizontal line) indicate no change compared with ancestral conditions. Values below 1 indicate lower fitness in selective media, that is a fitness cost. Each black point represents a single measurement. (*B***–***E*) Mean relative fitness of replicate populations in the four selective environments. Shaded areas represent the 95% confidence intervals for each replicate population. One EtOH population went extinct in the first 100 generations. Type II ANOVA and Tukey HSD tests; *n* = 118 for NaCl, 236 for other environments, ***P < 0.001; ns = not significant.

To test the expectation that more stressful environments lead to faster rates of adaptation, we assessed the fitness cost of each environment by comparing the fitness of the founder populations in each selective environment to their fitness in ancestral conditions (synthetic complete [SC] media). As a proxy for fitness, we used optical density (OD_600_) after 24 h in culture to estimate the cell density (supplementary table S1). Selective environments significantly differed in how much they reduced the fitness of the founder populations (fig. 2*A*; sodium chloride [NaCl] founder: *F*_118_ = 3826.2, *P* < 0.001; lithium acetate [LiAc] and EtOH founder: *F*_236_ = 286.7, *P* < 0.001). NaCl 0.75M (*t* = 61.9, df = 118, *P* < 0.001) and LiAc 0.02 M (*t* = 25.7, df = 236, *P* < 0.001) had the highest fitness costs. LiAc 0.01 M showed a more modest cost (*t* = 5.5, df = 236, *P* < 0.001). EtOH 8% had no significant impact on fitness (*t* = 1.1, df = 236, *P* > 0.05).

Next, we tested if evolving populations increased in fitness in their selective environments over time. Variation in fitness between replicate populations within environments was mainly explained by an interaction between replicate and generation, that is, fitness showed different trajectories over time in different replicates (fig. 2*B*–*E*, all *F* > 3.48, *P* < 0.001; df = 21, 308 for NaCl; df = 23, 314 for EtOH; and df = 24, 375 for both LiAcs). By the end of the experiment (700 generations for LiAc 0.01 M, LiAc 0.02 M and NaCl R1 and R4, and 1,000 generations for EtOH and NaCl R2 and R3), all replicate populations in the NaCl, LiAc 0.01 M, and LiAc 0.02 M environments had significantly higher fitness than the founder (all pairwise Tukey Honest Significance Difference [HSD] tests *t* > 3.48, *P* < 0.01, df = 308 for NaCl, df = 375 for LiAc 0.02 M, df = 300 for LiAc 0.01 M). On average, fitness increased by 118% in NaCl, 14.7% in LiAc 0.01 M, and 103% in LiAc 0.02 M. Replicate populations adapting to the two most stressful environments (NaCl and LiAc 0.02 M) had significantly higher fitness than the founder from the earliest time-point tested (generation 100) onwards (*t* > 2.78, *P* < 0.05). This was not the case for populations evolving in the lower stress environments (EtOH and LiAc 0.01 M). Three of the four replicate populations evolving in EtOH did not significantly differ from the founder at the end of the experiment (R1, R3, and R4: *t* < 2.43, df = 314, *P* > 0.1; R2: *t* < 6.42, df = 314, *P* < 0.001). In general, phenotypic divergence between replicate populations within each environment increased at later stages of evolution (mean of 15.8% significant pairwise Tukey HSD tests between replicates at 100 generations vs. 48.3% at 700 generations for all possible comparisons).

Averaged across replicates, the largest single increase in fitness occurred between 0 and 100 generations in three of the four environments (percentage of total fitness gains were 75.9% in NaCl, 89.3% in EtOH, and 100% in LiAc 0.02 M; fig. 2*B*–*E*). In LiAc 0.01 M, populations only reached 24.14% of their total fitness gain by 100 generations.

When tested in ancestral conditions (SC medium), evolved populations showed significantly higher fitness than the founder populations by the end of the experiment, although the magnitude of increase was small for LiAc environments (*F*_21,718_ = 251.77, *P* < 0.001; supplementary fig. S1, Supplementary Material online). In ancestral conditions, NaCl populations showed the largest increase in relative fitness within the first 100 generations, whereas populations evolved in EtOH increased only in later generations. This increased fitness could indicate adaptation to the SC component of the stressful environment (Johnson et al. 2021) or suggests that alleles providing high fitness in stress environments are also beneficial in ancestral conditions.

### Standing Genetic Variation Dynamics

**Fig. 3.**
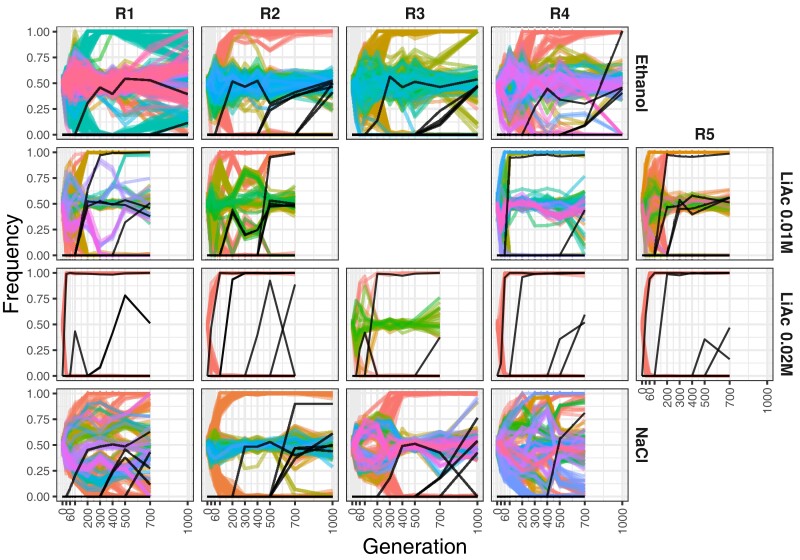
Frequency trajectories of variation during evolution. Haplotype clusters of standing genetic variation (in color) and high-frequency de novo mutations (black) across environments (rows) and replicates (columns). The allele frequency of the haplotype clusters is based on the SK1 allele. Note that a given haplotype might include multiple chromosomes.

In order to make comparisons between replicate populations and environments, we selected a set of 10,307 single-nucleotide polymorphisms (SNPs) originally present in the two parental strains and shared across all samples (i.e., no missing data; see supplementary fig. S2, Supplementary Material online for distributions of depth of coverage per sample). As expected from genetic drift and selection, we observed a tendency of alleles to either go to fixation or extinction as time progressed in the experiment (supplementary figs. S3–S6, Supplementary Material online). Typically a large proportion of ancestral variants settled into intermediate frequencies (around 0.5), suggesting the fixation of a single genotype that is heterozygous for those variants. Sets of variants shared similar allele frequency trajectories, likely due to genetic draft on a single or a few haplotypes (supplementary figs. S3–S6, Supplementary Material online). Hence, we reduced the 10,307 SNPs to sets of correlated variants or clusters in order to represent the overall ancestral variation dynamics (fig. 3). These clusters were constructed by correlating the normalized allele frequencies of all SNPs across all sampled generations following Otte and Schlötterer (2021). The correlation coefficients were transformed into a distance matrix and subject to average linkage hierarchical clustering using a range of minimum correlation coefficient values that maximized cluster number (see Materials and Methods). The result is a rough approximation of the number of haplotypes segregating per population. As we evolved F2 genotypes that underwent only one round of recombination, genetic linkage is high across the entire genome and increases during the whole experiment as any sweeps reduce genetic variation across the genome. Thus, a given “haplotype” cluster might contain variants from different chromosomes. However, note that real, not reconstructed, haplotypes within the population likely contain both Y55 and SK1 alleles, which may result in a “mirroring” of allele frequency trajectories, due to an increasing Y55 allele appearing as a decreasing SK1 allele, and vice-versa (fig. 3; supplementary figs. S3–S6, Supplementary Material online). Thus, when present in our clustering analysis, the mirror effect suggests that a single real haplotype of mixed ancestry swept through the population (see e.g., replicates 1 and 2 of LiAc 0.01 M in fig. 3).

Among all environments, LiAc 0.02 M showed the most drastic changes in allele frequencies across the genome (fig. 3; supplementary figs. S3–S6, Supplementary Material online). Specifically, in four out of five LiAc 0.02 M replicates, we observed that the standing genetic variation was depleted extremely quickly, leaving no intermediate allele frequencies. This pattern is consistent with the fixation of a single dominant genotype that was haploid or a fully homozygous diploid. In line with this interpretation, polymerase chain reaction (PCR) amplification recovered only a single mating type (*MATα*) from generation 30, the earliest time-point tested, in all replicates apart from replicate 3. It is possible that a few unmated cells (haploid products of F1 meiosis) survived at low frequencies in our F2 founding populations, which then had high fitness in LiAc 0.02 M. It is also possible that haploid cells underwent endoreduplication (Harari et al. 2018) in our experiment, restoring diploidy while retaining a single mating type. In any case, all heterozygosity was lost in these populations.

Surprisingly, the direction of change in the allele frequency of some variants in several populations shifted over the course of the experiment (fig. 3; supplementary figs. S3–S6, Supplementary Material online). In extreme cases, some alleles approached fixation at a given time point but then returned to intermediate frequencies (e.g., in the replicate 2 of LiAc 0.01 M; fig. 3). To further explore this pattern, we calculated the proportion of sites near fixation (minor allele frequency [MAF] ≤ 0.1) for each sample (at every time point of each population). We found that the proportion of nearly fixed sites at a given time point is not fully explained by the depth of coverage (supplementary fig. S7, Supplementary Material online), suggesting that the “unfixing” of sites is not an artifact of sequencing. However, some drastic frequency changes did coincide with the introduction and rise of de novo mutations (black lines in fig. 3), which in principle could change the direction of selection for the haplotype containing them (see next section for further discussion).

**Fig. 4.**
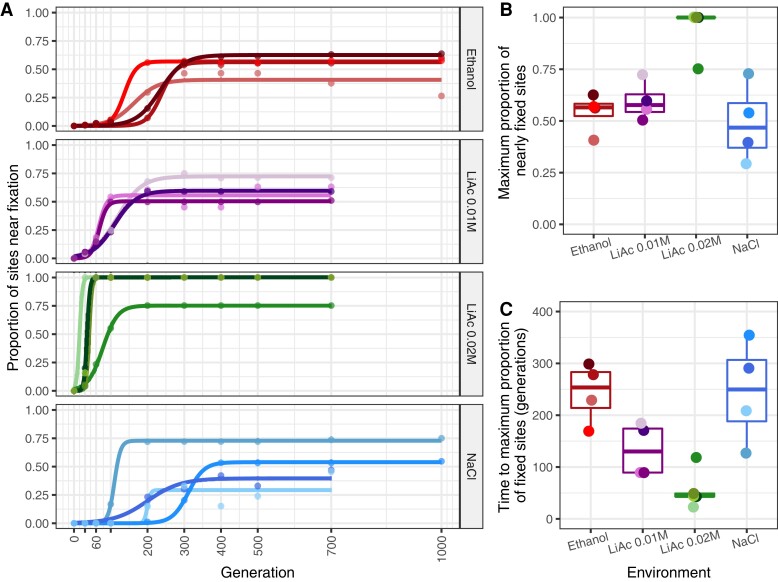
Differences in the proportion of nearly fixed sites (MAF ≤ 0.1) across environments. (*A*) The raw proportion of nearly fixed sites through time (points) were fitted to a sigmoidal model for each replicate, (*B*) each with their respective maximum proportion of nearly fixed sites, and (*C*) the time at which each replicate reaches this maximum. There are significant differences between environments with respect to the maximum proportion of nearly fixed sites (Kruskal–Wallis H_3_ = 10.549, *P* = 0.014, *n* = 17) and time to the maximum proportion of nearly fixed sites (Kruskal–Wallis H_3_ = 11.325, *P* = 0.010, *n* = 17), but pairwise Wilcoxon tests were not significant after Bonferroni correction.

Regardless of the drastic changes of some haplotypes, a considerable fraction of variants reached fixation in all populations (at generation 700, a fraction of 0.37–0.63 sites in EtOH; 0.45–0.73 in NaCl; 0.51–0.71 in LiAc 0.01 M; 0.75–1.00 in LiAc 0.02 M). Fitting a sigmoidal model to the proportion of nearly fixed sites in each replicate population shows that the curves often plateau at proportions close to 0.5, 0.75, or 1 (fig. 4*A*). A proportion of 0.5 is expected if a single F2 genotype was fixed in a given population, as their inbreeding coefficient is 0.5 by experimental design (equivalent to one round of intertetrad mating; Kirby 1984). The proportion of 1 is consistent with a single haploid or fully homozygous diploid going to fixation. The origin of the 0.75 fraction is less clear. Overall, the maximum proportion of nearly fixed sites differs between environments (Kruskal–Wallis H_3_ = 10.549, *P* = 0.014), mostly due to the complete fixation of alleles in the LiAc 0.02 M environment (fig. 4*B*). Plateauing also occurred faster in LiAc 0.02 M, typically within the first 100 generations, than in any other environment (fig. 4*C*; Kruskal–Wallis H_3_ = 11.32, *P* = 0.010). These observations are consistent with stronger selection in LiAc 0.02 M. Although the NaCl environment also imposed high fitness costs (fig. 2*A*), there was large variation between replicates in the proportion of nearly fixed sites, and diversity was generally lost at a slower pace than in the other environments (fig. 4*C*; but pairwise Wilcoxon tests were not significant after Bonferroni correction). Likewise, many distinct haplotypes were reconstructed in replicates 1 and 4 of the NaCl environment (fig. 3), suggesting that multiple genotypes persisted for longer in those populations.

**Fig. 5.**
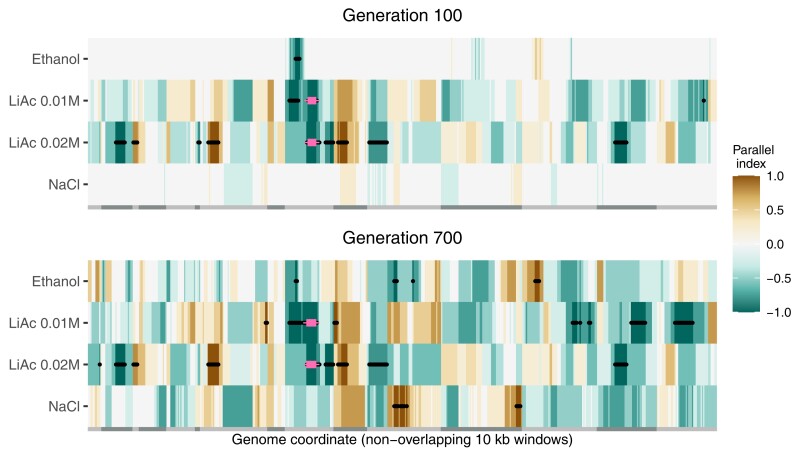
Heatmaps of genetic parallelism along the genome in the different environments. The genome was divided into non-overlapping 10 kb windows with at least four SNPs and the median allele frequencies were used to calculate a parallelism index. The index is a scaled count of how many replicates are fixed (MAF ≤ 0.1) for the SK1 parental allele (+1) or the Y55 parental allele (−1). Chromosome limits are defined by alternating light and dark gray bars at the bottom of the heatmaps. Black points mark windows where either parental allele was fixed in four or more replicates (LiAc 0.02 M has five replicates, all other environments had four). Pink crosses mark windows that were completely fixed in all the LiAc replicates (both LiAc 0.01 M and LiAc 0.02 M).

In order to explore parallelism at the genetic level, we divided the genome into non-overlapping 10 kb windows and calculated an “environment parallelism” index that ranged from 1 (all replicates fixed the SK1 allele) to −1 (all replicates fixed the Y55 allele) (see Materials and Methods). The resulting heatmap in figure 5 revealed strong parallelism for the LiAc environments but little to none in the other two environments early in the experiment (generation 100). Excluding NaCl, the number of windows fixed for a parental allele followed the stress gradient, that is, LiAc 0.02 M had the most windows, followed by LiAc 0.01 M, and then EtOH (supplementary fig. S8, Supplementary Material online). Most notably, a region in chromosome 9 was consistently fixed for the Y55 allele in all replicates of the LiAc environments (red points in fig. 5). This region (chromosome 9: 200,001–270,001) contains about 39 genes, but no obvious causative variant that might drive adaptation to LiAc. As expected from the haplotype trajectories (fig. 3), this same region was already fixed in the haploid-like LiAc 0.02 M replicates from generation 30 (supplementary fig. S9, Supplementary Material online). An adjacent region to the left of the chromosome was also already fixed for the Y55 allele in multiple replicates of both LiAc environments by generation 30 and partially in EtOH (supplementary fig. S9, Supplementary Material online), suggesting it might be more generally adaptive to the basic SC growth medium (or that the SK1 allele is generally deleterious and went to extinction). Toward the end of the experiment (generation 700), all environments exhibited at least a few windows with high parallelism (fig. 5 and supplementary fig. S8, Supplementary Material online).

Given the frequency trajectories of the populations in NaCl, we can distinguish two types of replicates at generation 700: populations who were dominated by a single diploid genotype (R2 and R3) and populations who maintained more diversity (R1 and R4). Interestingly, populations within each type fixed the same allele in 25.6% of analyzed windows (129 out of 504). In contrast, only 3.2% (16 windows) overlapped between the two population types (fig. 5; supplementary fig. S10, Supplementary Material online). For comparison, two randomly selected F2 siblings should share 63 windows by chance (see Materials and Methods). With the caveat that there are only two replicates in each group, the fact that the two types of populations have such different sets of fixed windows suggest that at least two sets of multiple alleles contributed to NaCl adaptation—a pattern that fits well with what is expected under polygenic adaptation with genetic redundancy (Barghi et al. 2020).

### De Novo Mutations Dynamics

From the detected 323 putative de novo mutations (190 SNPs and 133 insertions/deletions [INDELs]), we focused on potentially adaptive variants that reached a frequency >35% during at least one time point in any sample of the experiment (56 SNPs and 6 INDELs after additional manual curation as described in the Materials and Methods; black lines in fig. 3). The 35% threshold represents an arbitrary cutoff to exclude sequencing and variant calling errors, while still detecting mutations that might be adaptive but arose late in the experiment. The SnpEff program predicted moderate-to-high fitness effects for these high-frequency variants, as expected if they were under selection (supplementary fig. S11, Supplementary Material online). It is worth noting that, in a diploid asexual population, a new mutation should reach a maximum frequency of 0.5 (i.e., in a heterozygous state). The fact that many de novo mutations reach frequencies of 1 (fig. 3) implies some mechanism of gene conversion to become homozygous, as commonly observed in other yeast studies (Vázquez-García et al. 2017; Johnson et al. 2021).

**Fig. 6.**
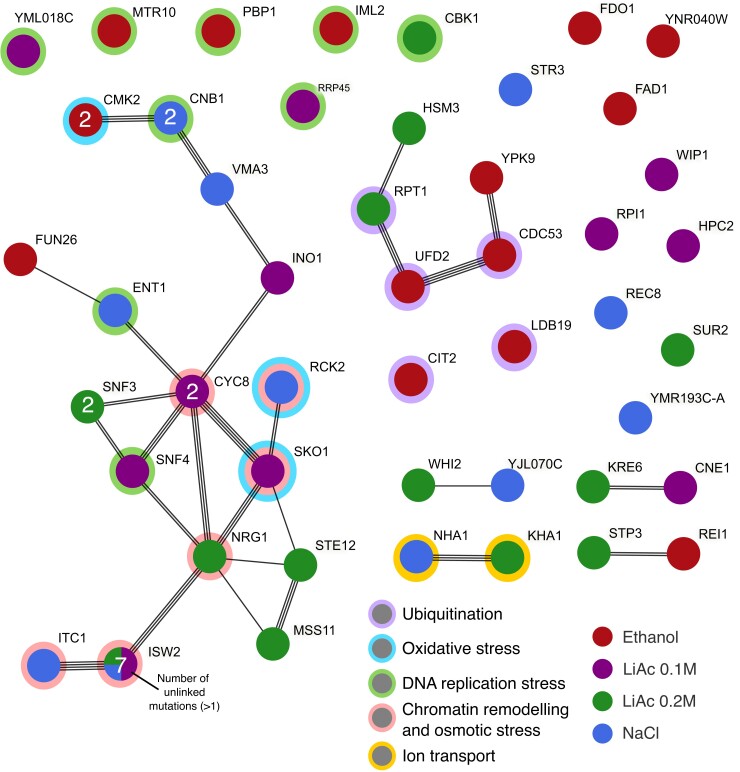
Protein–protein interaction network of all genes affected by de novo mutations that reached a frequency of at least 35% at some point during the experiment. Each node is a pie chart of the proportion of mutations per gene that occurred in each environment (all present in a single environment, except for *ISW2*). The number of unlinked independent mutations is indicated within each node if a gene was hit by more than one mutation (two mutations in the genes *SUR2* and *SNF3* have linked allele frequency trajectories and were counted as one). The links represent different sources of interaction evidence detected by the STRING database. Main functional categories are depicted by rings around the nodes.

**Fig. 7.**
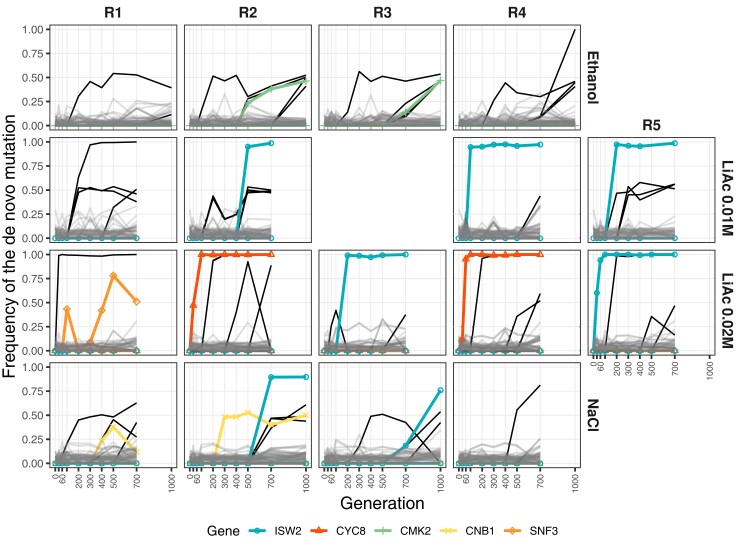
Genes with parallel independent de novo mutations. Of the de novo mutations that reached a frequency >35% at some time point (black lines), some hit the same gene independently (in colors). Mutations that stayed below 35% frequency are presented in gray.

Most of the high-frequency de novo mutations were located within protein-coding regions (57 out 62), affecting in total 47 genes with 6 synonymous mutations, 35 nonsynonymous point mutations, 6 frame-shift mutations, 9 stop codon gains, and 1 start codon loss. Notably, a STRING network of these 47 genes has significantly more interactions than expected given a random set of proteins of the same size and node degree distribution (protein–protein interaction enrichment *P* = 0.00156; Szklarczyk et al. 2021; fig. 6). In particular, multiple independent mutations hit members and known cofactors of the protein complexes Isw2p-Itc1p and Cyc8(Ssn6)p-Tup1p in almost all replicates of the NaCl and LiAc environments (red rings in fig. 6), reaching frequencies of around 0.5 (expected if a single heterozygous genotype fixed) or 1 (fig. 7). The transcriptional corepressor complex Cyc8p-Tup1p recruits the chromatin remodeling complex Isw2p-Itc1p to nucleosomes, controlling the repression of hundreds of genes under stressful conditions (Green and Johnson 2004; Zhang and Reese 2004; Rizzo et al. 2011). Many of these genes are activated in response to cellular stress, including osmotic stress (Proft and Serrano 1999; Proft and Struhl 2002; Green and Johnson 2004).

In our experiment, the gene *CYC8* (YBR112C or *SSN6*) gained two independent missense mutations in different replicates of LiAc 0.02 M (fig. 6). Notably, it has been shown that the Cyc8p*-*Tup1p complex acts as a repressor of *SKO1* (YNL167C), a gene that gained an internal stop codon in one replicate of LiAc 0.01 M (fig. 6). *SKO1*, in turn, is a downstream effector of the high-osmolarity glycerol (HOG) pathway, which activates during osmotic stress and glucose starvation (Proft and Serrano 1999). Along with the Cyc8p-Tup1p complex, Sko1p represses the primary sodium and lithium-ion transporter *ENA* (Proft and Serrano 1999). Likely, deactivation of these members of the HOG pathway makes the expression of *ENA* constitutive (at least to a degree, see Proft and Serrano 1999), allowing for the constant expression of the genes involved in tolerating osmotic stress. Moreover, Sko1p is involved in recruiting other salt stress defense genes. Accordingly, deletions of *SKO1* have been shown to provide resistance against Na^+^ or Li^+^ (from NaCl and LiCl) stress (Proft and Serrano 1999).

It is unknown if Isw2p-Itc1p interacts with Cyc8p-Tup1p while specifically repressing *SKO1*, but this is a strong possibility given that their effects on nucleosome positioning is highly correlated across the genome (Rizzo et al. 2011). Remarkably, up to seven independent de novo mutations disrupted the gene *ISW2* (YOR304W) in multiple environments (fig. 7), including the loss of the start codon, gains of stop codons, and frame shifts. Likewise, one additional frame-shift INDEL affected the gene *ITC1* (YGL133W) in a NaCl population. Together, Isw2p-Itc1p is also known to repress early meiotic genes during mitotic growth (Goldmark et al. 2000). The de-repression of these early meiotic genes by deleting *ISW2* does not cause slow growth in cells (Goldmark et al. 2000). Hence, disruption of this protein complex might be advantageous during asexual reproduction under osmotic stress.

Similarly, in the NaCl environment, two nonsynonymous mutations affected the gene *CNB1* (figs. 6 and 7) that codes for the regulatory subunit of calcineurin, a regulator of *ENA* transcription in response to sodium and lithium toxicity (Mendizabal et al. 1998; Ruiz et al. 2003). Thus, the regulation of *ENA* seems to be a common denominator in the molecular basis of adaptation to osmotic stress in our experiment. In nature, the ENA gene can exist in one or multiple copies depending on the strain of yeast (Rodríguez-Navarro et al. 1994; Ruiz et al. 2003), and specific paralogs and copy number variation have phenotypic effects on sodium and lithium sensitivity (Rodríguez-Navarro et al. 1994; Warringer et al. 2011). In the strains ancestral to our experiment, SK1 and Y55, there is a single ENA copy in chromosome IV. Hence, we compared the relative depth of coverage of the ENA gene against the flanking regions in the same chromosome (about 100 kb on each side), with the expectation that drastic copy number changes that fix in a population should lead to significant deviations in ENA's coverage. However, we found no evidence of changes of copy number variation in any population (supplementary fig. S12, Supplementary Material online).

In addition to genes controlling the expression of ENA, other genes affected by mutations in the NaCl and LiAc environments have functions associated with oxidative stress, ion transport, and DNA replication stress (fig. 6). In contrast, the high-frequency mutations found in the EtOH environment had less connections to those found in other environments, and were mostly associated with ubiquitination and DNA replication stress functions (fig. 6), which is in line with previous molecular characterization of the ethanol (EtOH) stress response (Stanley et al. 2010; Navarro-Tapia et al. 2018).

The trajectory of some mutations on their way to fixation changed abruptly. In fact, some mutations went extinct, during the time when the frequency of another mutation increased in the population (e.g., replicate 2 of LiAc 0.02 M or replicate 3 of NaCl in fig. 7), in a pattern reminiscent of clonal interference (Gerrish and Lenski 1998). This putative turnover of genotypes happened after the standing genetic variation was completely sorted in some populations, but it might have also affected the fate of standing genetic variation (e.g., replicate 2 of LiAc 0.01 M in fig. 3). Although it is tempting to speculate that some radical changes in the direction of a particular haplotype were due to the evolution of adaptive mutations, some mutations may in fact be neutral or deleterious and increased in frequency through genetic hitchhiking on a haplotype affected by other processes (e.g., cryptic fluctuating selection during the experiment). However, the fact that multiple high-frequency independent mutations hit the same genes in different replicates argues for their adaptive value (Kryazhimskiy et al. 2014). Specifically, the genes *CMK2*, *CNB1*, *CYC8*, and *SNF3* all had two unlinked mutations in two different replicates of different treatments, whereas the gene *ISW2* had seven (fig. 7), as mentioned above. The probability of multiple independent mutations affecting these genes is very low (*P* < 0.005, see Supplementary materials), and vanishingly small for *ISW2* in particular (*P* = 1.39 × 10^−19^; supplementary table S2, Supplementary Material online).

### Chromosomal Copy Number Changes

**Fig. 8.**
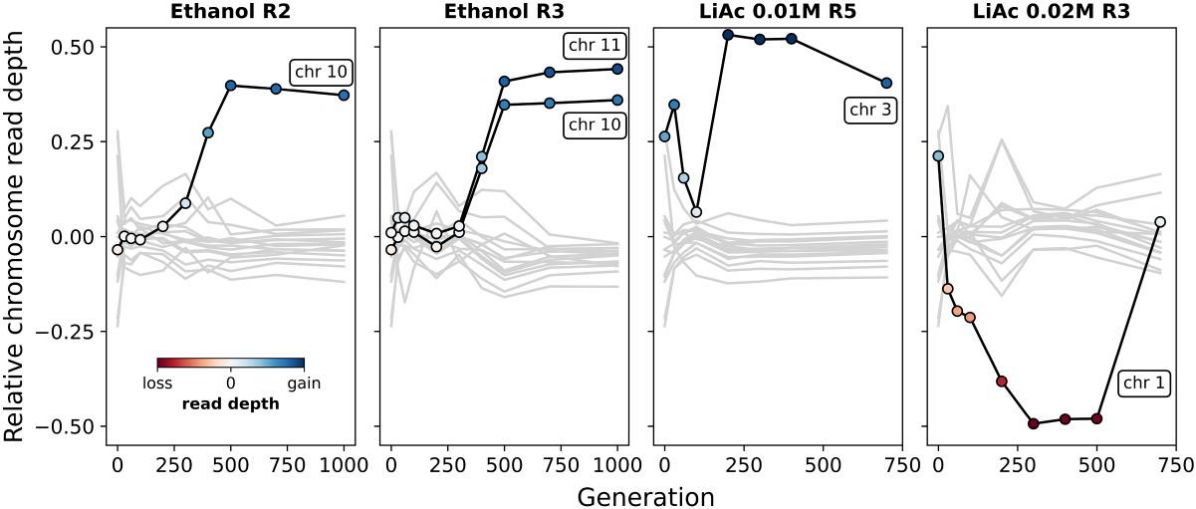
Chromosome copy number changes. A subset of chromosome copy number changes in four replicate populations. Lines show the relative read depth of each chromosome (read depth vs. the mean read depth across all chromosomes). Lines in black indicate chromosomes with potential aneuploidies. The points within indicate a gain (blue) or loss (red) of chromosome, with the shade indicating the magnitude of relative read depth change.

Using read depth, we found that changes in chromosomal copy number within the evolving populations were common over the course of experimental evolution. Thirty-five percent (6 of 17) of all replicate lines had at least one chromosome that deviated from the genome-wide mean read depth by >25% for at least one time point (supplementary fig. S13 and table S3, Supplementary Material online). This was dominated by chromosome gains (81%) as opposed to losses (19%). Although some of these potential aneuploidies were limited to single time points, we found several cases where aneuploidies were maintained in the evolving populations across hundreds of generations (fig. 8). For instance, in EtOH, we detected the same aneuploidy (2× chromosome 10 and 1× chromosome 11) in at least three subsequent time points spanning 500 generations, which were maintained in the populations until the end of the experiment. One notable example of parallel patterns in copy number variation is chromosome 10 in EtOH which significantly increased in relative chromosome read depth in two independent replicates (fig. 8). No significant deviations were found in the NaCl environment. In addition, none of the de novo mutations that went to full fixation (frequency of 1) coincide with detected chromosome changes, suggesting that the selective advantage of the detected aneuploidies might depend on the standing genetic variation or on intrinsic properties that come with ploidy change (e.g., gene dosage). Furthermore, it also implies that other mechanisms, such as gene conversion and mitotic recombination, were involved in the fixation of de novo mutations that appeared in a diploid background.

## Discussion

Although adaptation from standing genetic variation is an important process underlying evolution in natural populations, we are often unable to characterize the timing and dynamics of fitness changes, and the underlying genetic mechanisms. Here, we used microbial experimental evolution and whole-population sequencing to track the phenotypic and genomic changes of genetically diverse yeast populations in environments with different stress levels. To simulate adaptation dynamics in more natural demographic conditions, we used outcrossed founder populations containing more standing genetic variation than the isogenic starting populations traditionally used in microbial experiment evolution. As expected, populations increased in fitness over time and showed significantly larger fitness gains in more stressful environments. The genetic diversity present in the founder was rapidly sorted and decreased quickly in most but not in all environments over time. Surprisingly, some populations of NaCl in particular maintained multiple genotypes until the end of experimental evolution without the majority of alleles getting fixed, despite high-stress levels. We detected parallelism at both phenotypic and genotypic levels (involving genes, pathways, and aneuploidies) within and between environments, with conspicuous changes recurring in the high-stress environment LiAc 0.02 M. In summary, our results suggest that adaptation was driven by both standing genetic variation and de novo mutations in our experiment, with interesting environmental idiosyncrasies.

### Adaptation Dynamics in Stressful Environments

In previous experiments with similar starting conditions (i.e., outbred founding populations and asexual reproduction) fitness gains quickly reached a plateau during evolution (e.g., Kosheleva and Desai 2018). Accordingly, we observed the strongest increases in fitness (>100%) in the two environments with the high fitness cost (NaCl and LiAc 0.02 M in fig. 2) but found no or low fitness increases in the two less stressful environments. Most of the fitness increase occurred early in the experiment (in the first 100 generations), likely due to the sorting of the genetic diversity present in the founder. These fitness patterns are consistent with declining adaptability and diminishing returns of beneficial variants that have consistently smaller effects in fitter backgrounds (Wiser et al. 2013; Kryazhimskiy et al. 2014; Johnson et al. 2019). Specifically, populations adapting to NaCl and LiAc 0.02 M were further away from their fitness optimum, leaving more possibility for fitness improvements. However, it is also important to note that our OD_600_ measurements only capture the endpoint of a complex growth cycle. Fitness in serial transfer experiments is integrated over several complex phases of growth, such as lag, fermentation, respiration, and stationary phase (Li et al. 2019). It may be the case that phenotypic adaptation occurred in phases that do not contribute as heavily toward the final OD as the exponential fermentation phase and were thus missed by our endpoint estimates.

By the end of the experiment many populations were seemingly dominated by a single diploid genotype, typical of populations evolving under strict asexual reproduction (Kosheleva and Desai 2018). The number of nearly fixed sites and the speed at which these sites reached near fixation increased with the stress level of the environments (fig. 4). One exception here is the NaCl environment, where we found more variation between replicates, alleles took a longer time to fixation, and the number of alleles nearing fixation was lower than in the other high-stress environment. Likewise, early in the experiment, there were multiple regions fixed independently for the same genotype in the LiAc environments and at least one in EtOH, but none in NaCl despite high phenotypic parallelism. It is possible that this reflects the complexity of the trait, suggesting that adaptation to salt is polygenic and involves many loci of small effect with interactions between them, in agreement with previous studies (Dhar et al. 2011). On the other hand, de novo mutations occurred in similar pathways in both NaCl and LiAc environments, suggesting considerable overlap in target loci. Although not directly observable with the structure of our data, the trajectories of some de novo mutations are consistent with clonal interference (Gerrish and Lenski 1998), as observed in other long-term experiments with yeast (Johnson et al. 2021).

### Adaptation to High Concentrations of Lithium Acetate is Characterized by Fast Evolution of Functional Haploidy

Of the environments investigated, LiAc 0.02 M had the most conspicuous changes: four out of five populations were depleted of all standing genetic variation at a remarkably fast pace, likely through a transition to haploidy or fully homozygous diploidy, and always with a *MATα* mating type. The prevalence of the same mating type in this environment may indicate higher fitness of *MATα* (which came from the SK1 parent) or any variant linked to it. Alternatively, the fixing of the *MATα* allele across replicates was a chance event (binomial probability of 0.156).

As none of the LiAc 0.01 M populations showed an equivalent response, the fast depletion of diversity in LiAc 0.02 M might indicate that this is a dose-dependent response to LiAc. Previous studies have shown that adaptation is typically faster in haploids than in diploids under large population sizes (Zeyl et al. 2003; Johnson et al. 2021). This is because mutations are readily exposed to selection in haploids regardless of their dominance coefficient, and there are multiple other ploidy-dependent effects that can make haploidy advantageous, such as protein-dosage changes and cell size (discussed by Gerstein et al. 2011). Although our PCR mating type test identified that only the MATα mating type was present, this does not differentiate between haploidy and homozygous diploidy. In either case, the loci involved might not be as advantageous in a heterozygous state. We note, however, that the fitness of the one population (replicate 3) that remained diploid was just as high as that of the other populations, suggesting that functional haploidy is not the only route for adaptation in LiAc 0.02 M. Haplotype trajectories in this replicate are consistent with the fixation of a single diploid genotype, with an overall loss of 75% of the standing genetic variation (figs. 3 and 4). Ultimately, dissecting the genetic basis of the response to LiAc will be necessary to explain these patterns.

### Parallelism at the Phenotypic and Genomic Level

At the phenotypic level, we observed similar fitness responses within environments, especially at the earliest time point measured (generation 100), and most conspicuous in the high-stress environment NaCl and LiAc 0.02 M (fig. 2*A*). This suggests that phenotypic parallelism was primarily driven by standing variation. After this, replicate populations diverged significantly in fitness (fig. 2*B*–*D*). This is interesting because most populations were already fixed for a single genotype at this point, which suggests that it was the influx of de novo mutations that diversified phenotypic evolution. In addition, similar fitness values at early stages of evolution might result from genetic redundancy, with multiple subsets of small-effect loci contributing to adaptation but leading to the same phenotype (Barghi et al. 2019). This last scenario might apply to some NaCl populations, where the phenotypic response was highly parallel but multiple genotypes persisted even after 700 generations (fig. 3) and no region was consistently fixed for a parental genotype early in the experiment in all populations (fig. 5, supplementary fig. S10, Supplementary Material online).

Notably, adaptation to either concentration of LiAc was characterized by the fixation of a single Y55 haplotype in chromosome 9, but other genomic regions fixed in parallel were different between LiAc 0.01 M and LiAc 0.02 M (fig. 5). Thus, the LiAc 0.01 M environment is not simply a delayed version of LiAc 0.02 M where the same variants fix at a slower pace. Instead, at least one locus is generally beneficial when exposed to LiAc, but other combinations of parental loci are advantageous under different concentrations.

Parallelism at the gene and pathway level due to de novo mutations is often observed in experimental evolution of bacteria and yeast (Tenaillon et al. 2012; Kryazhimskiy et al. 2014; Huang et al. 2018; Harris et al. 2021). Here, we detected multiple mutations in genes associated functionally to the HOG pathway, in particular through the Cyc8p-Tup1p and Isw2p-Itc1p complexes (fig. 6). Interestingly, we also observed parallelism *across* environments, NaCl and the two concentrations of LiAc, most notably through multiple mutations disturbing the *ISW2* gene. As NaCl and LiAc are both salts, they likely exert similar selective pressures in the shape of, for example, osmotic stress. Nonetheless, we saw no common tracks of standing genetic variation being fixed in parallel between the NaCl and the LiAc environments. These two observations, the similarity in de novo mutations but the different parallelism index of standing genetic variation, seem to be in contradiction. However, it is possible that NaCl and the LiAc environments are different enough, and that the parallel response in de novo mutations reflects the fact that the genes involved in the stress response system of *S. cerevisiae* are highly pleiotropic. For example, in addition to the stress response, *ISW2* is required for early stages of sporulation (Trachtulcová et al. 2000) and the silencing of subtelomeric genes (Iida and Araki 2004). Likewise, although the HOG pathway gets activated by osmotic stress, it is also important for the response to heat and oxidative stress, among other stressors (Hayashi and Maeda 2006; Hohmann 2009). In an experiment to adapt *S. cerevisiae* to increasing temperature, Huang et al. (2018) detected de novo mutations affecting Swip/Snfp, another chromatin remodeling complex heavily involved in multiple stress responses. Swip/Snfp happens to be recruited by the product of *SKO1*, one of the genes mutated in our experiment and repressed by Cyc8p-Tup1p (Proft and Serrano 1999). Moreover, independent populations of the NaCl environment and in the temperature treatment of Huang et al. (2018) acquired mutations affecting members of the calcium-calcineurin pathway (*CNB1* in our case and *CRZ1* in theirs), once again highlighting the high level of pleiotropy associated with the yeast stress responses.

In contrast, in the EtOH environment, we observed mutations affecting a different set of genes implicated in other processes, in particular ubiquitination. It has been shown that EtOH can partially repress the HOG pathway activation (Hayashi and Maeda 2006), implying that the physiological response necessary for EtOH adaptation might be very different to that of NaCl, LiAc, and even heat. Notably, when comparing the dynamics of standing genetic variation, one specific locus was fixed in parallel in both the NaCl and EtOH environments, but with opposite parental alleles (middle of fig. 5 at generation 700). Future research could center on potential trade-offs between adaptation to EtOH and the other environments.

In addition to de novo point mutations and small INDELs, we also detected cases of parallelism in chromosome copy number variation (fig. 8, supplementary fig. S13, Supplementary Material online). Aneuploidies of chromosome 10 in EtOH were maintained for hundreds of generations in two replicates. Aneuploidies are costly to maintain as they can lead to reduced rate of cell division and cause havoc in gene expression (Torres et al. 2007; Pavelka, Rancati, and Li 2010; Pavelka, Rancati, Zhu, et al. 2010; Sheltzer et al. 2011; Dürrbaum and Storchová 2016). However, there is increasing evidence that aneuploidies can facilitate adaptation to environmental stress in yeast (reviewed in Gilchrist and Stelkens 2019) and drive fitness increases in laboratory-based studies (Selmecki et al. 2009; Chen et al. 2012; Millet et al. 2015; Lauer et al. 2018) and wild populations (Gasch et al. 2016; Tsai and Nelliat 2019; Scopel et al. 2021). As aneuploidies are often costly, the temporal patterns and parallelism we observe across replicates suggest they were advantageous enough to be maintained.

We detected no direct link between the distribution of de novo mutations and aneuploidies, suggesting that this parallelism is driven by genetic variation that pre-existed in the founder, and became adaptive through changes in copy number. On the other hand, the average beneficial fitness effects of aneuploidies might be larger in general than those of de novo mutations (SNPs or INDELs). Thus, aneuplodies might rise in frequency more easily, overwhelming any alternative adaptive de novo mutations appearing in the population.

Overall, we see evidence of extensive parallelism at the phenotypic and molecular level derived from both standing genetic variation and de novo mutations. Although we are limited by the resolution of a single recombination event in our founder populations, we were able to infer some aspects of the genetic architecture underlying adaptation in the chosen environments: a potential large-effect locus in the LiAc treatments, polygenic adaptation in general and genetic redundancy in NaCl in particular, and multiple mutations affecting the HOG pathway.

### Interaction Between Standing Genetic Variation and de novo Mutations

One unexpected aspect of our data is the changing trajectory of some haplotypes, most notably in LiAc 0.01 M populations, but also in some NaCl populations (fig. 3). We found little evidence that methodological artifacts, namely low depth of coverage, explain this pattern. However, we did observe simultaneous emergence of de novo mutations that follow the trajectory of changing haplotypes of standing genetic variation. Just like mutations can compete in otherwise isogenic populations (i.e., clonal interference), these mutations might have changed the trajectory of resident haplotypes to the detriment of some others, thus contributing to the maintenance of variation for longer periods.

Some of the unexpected changes in haplotype frequencies may also be explained by fluctuating selection caused by the evolving populations themselves, for instance through changes in cell metabolism impacting the pH of the growth medium, which may have altered the direction of selection for some haplotypes. Changes in the growth medium may affect yeast populations in similar ways as seasonal changes cause trait and genomic shifts in populations of fruit flies as recently shown in an elegant field experiment, where the continuous adaptation in response to rapid environmental change was described as adaptive tracking (Rudman et al. 2022).

## Conclusion

Our study shows that adaptation dynamics in non-recombining systems are affected by an intricate interplay of standing genetic variation, de novo mutations, and different levels of environmental stress. Follow-up studies are needed to provide a better understanding of the relative roles that each of these factors, and their interactions, play in changing a population's adaptive potential to future environmental change. As we found considerable variation in population responses to different concentrations of one stressor (LiAc), one interesting angle would be to use an evolve-and-resequence approach along a fine-scaled environmental gradient of stress, to mimic population responses to gradual pollution events and environmental deterioration. Similarly, one could create a gradient of standing genetic variation, ranging from clonal to increasingly outbred populations, to conclusively test how increasing genetic diversity affects adaptation dynamics. Pairing experimental evolution with long read sequencing could be used to scrutinize the role of larger structural variants beyond small INDELs in adaptation dynamics (for instance transposable elements, inversions, and translocations), to conclusively test for their causality in fitness changes and adaptive processes. We hope our study contributes to a better understanding of the constraints and drivers shaping the evolutionary potential of populations and their responses to future environmental change.

## Materials and Methods

### Strain Engineering and Crosses

We made F1 crosses between *S. cerevisiae* strains SK1 (*MATα ho::KANMx*) and Y55 (*MATa ho leu2::HYGMx*) and sporulated them in 50 ml KAc media (1% potassium acetate, 0.05% glucose, 0.1% yeast extract made up to 1 l with sterilized, deionized H_2_O) to generate large, genetically recombined diploid founder populations with standing genetic variation. To be able to identify populations on dropout media, we transformed the original SK1 and Y55 strains to contain auxotrophies for genes that synthesize essential amino acids. Following the protocol by Gietz and Schiestl (2007), we knocked out the functional copies of either uracil (*ura3*) or lysine (*lys2*) and replaced them with a drug resistance gene (nourseothricin sulfate; clonNAT). We verified the successful insertion of all drug cassettes by PCR. Founding strains thus contain alternative antibiotic resistance markers and auxotrophies, but otherwise the same strain background. Markers KANMx and HYG were used to select for F1 offspring of the original SK1×Y55 cross. Specifically, the founders of populations evolving in NaCl are the diploid F2 offspring from a cross between SK1 (*MATα ho:KANMx ura3::ClonNat*) and Y55 (*MATa ho leu2::HYGMx ura3::ClonNat*). All other populations (LiAc 0.01 M, LiAc 0.02 M and EtOH) are the diploid F2 offspring of a cross between SK1 (*MATα ho:KANMx lys2::ClonNat*) and Y55 (*MATa ho leu2::HYGMx lys2::ClonNat*). Note that NaCl adapted populations lost their ura3 auxotrophy within the first 100 generations, whereas the other populations lost their G418 resistance encoded by the KANMx cassette. All other expected markers were maintained. The fitness of the founder populations was measured in selective environments (OD_600_ after 24 h in culture) and used to standardize the evolved populations’ fitness.

### Environments and Serial Transfer

All environments consisted of SC medium (2% glucose, 0.67% bacto-yeast nitrogen base without amino acids, 0.00079% Formedium CSM powder [product code DCS0019] made up to 1 l with sterilized, deionized water) plus a stress-inducing substance. We used four substances: LiAc 0.01 M, LiAc 0.02 M, NaCl 0.75 M, and EtOH 8%. Replicate populations were grown in separate 60 ml glass test tubes containing 5 ml of SC media with the appropriate stressor added. Every 48 h, after growth at 30 °C with shaking, 10 µl of culture (0.02%) was transferred to fresh media. This corresponded to around seven generations, estimated by counting cells after 48 h growth in selective media with 0.75 M NaCl. For the EtOH environment, the correct volume of EtOH was added to the tube just before inoculation to limit evaporation. Every five transfers (10 days, representative of ∼35 generations) 900 µl population samples were frozen in 900 µl of 44% glycerol and stored at −80 °C for further analyses. Populations were checked regularly for environmental contamination by microscopic inspection and plating out on agar. We also controlled for external fungal contamination using the selectable genetic markers. The experiment was run for a total of ∼300 days.

### Fitness Dynamics

We measured the fitness as OD_600_ after 24 h of growth in culture of all evolving populations at generations: 0, 100, 300, 500, 700, and 1000. All populations were measured in the environment they were selected in. Relative yield for adapted populations was calculated as ODadapted/mean(ODfounder) for each OD measurement taken. A value of >1 represents higher fitness than the mean founder population fitness. In addition, we measured populations in SC only, which is the nearest equivalent to the ancestral medium due to the laboratory-based history of the two founder strains SK1 and Y55. For further details on OD measurements, see Supplementary Materials.

Type II analysis of variance (ANOVA) and Tukey HSD tests were used to compare the fitness of evolved populations to the founder, as well as the founder's fitness in the selective environments to fitness in ancestral conditions. To explain variance in fitness between replicate populations, we used a linear model with generation, replicate and their interaction as fixed effects, selecting the simplest model using type I ANOVA. All statistical analyses and graphs were carried out with R v4.1.0 (R Core Team 2021), using the packages, car v3.0–5 (Fox and Weisberg 2019), dplyr v1.0.7 (Wickham et al. 2021), emmeans v1.7.1-1 (Lenth 2022), ggplot2 v3.3.5 (Wickham 2016), gridExtra v2.3 (Auguie 2017), and MASS v7.3-54 (Venables and Ripley 2002). Raw fitness data are available in supplementary table S1, Supplementary Material online. Scripts used for analyses and plotting are available at https://github.com/SLAment/AdaptationDynamics.

### DNA Extraction and Whole-Genome Sequencing

DNA was extracted from whole population samples at the nine time points (0, 30, 60, 100, 200, 300, 400, 500, 700, and 1,000 generations) of experimental evolution using Thermo Scientific KingFisher™ Duo Prime Purification, as per manufacturer's instructions (see Supplementary Materials for further details). For this, 10 µl of frozen populations were grown in 5 ml SC media at 30 °C shaking at 200 rpm for 24–48 h (until near saturation) and ∼1 ml of the culture was used for DNA extraction Library preparation followed the Illumina Nextera Flex protocol and sequencing was carried out by Illumina Pool-Seq using either the NovaSeq S4–300 (2 × 150 bp) or NovaSeq S-Prime (2 × 150 bp) to an estimated median depth of coverage per sample of around 30× to 780×. The raw sequences were deposited in the European Nucleotide Archive, accession number PRJEB46680. In addition, we included publicly available Illumina reads of the parental strains SK1 and Y55 in all analyses (accession numbers PRJNA340312 and PRJNA552112; Yue et al. 2017; Bendixsen et al. 2021).

### Quality Control, Mapping, and Variant Calling

Both FastQC v0.11.9 (Andrews 2010) and MultiQC v1.11 (Ewels et al. 2016) were used to assess the quality of the raw sequencing reads, both before and after trimming using Trimmomatic v0.36 (Bolger et al. 2014). Samples LiAc0.02 R2 (replicate 2) at 60 generations and LiAc0.01 R5 at 500 generations were dropped due to poor coverage and sequencing failure, respectively. The genome of strain S288C (R64; GenBank accession GCA_000146045.2) was used as the reference. We then carried out alignment using BWA v0.7.17 (Li and Durbin 2010) and samtools v1.9 (Li et al. 2009) to sort the generated bam files. Duplicated reads were removed using picard v2.23.4 (http://broadinstitute.github.io/picard/), followed by quality assessment of the mapping with QualiMap v2.1.1 (Okonechnikov et al. 2015) and MultiQC. To call variants, we used GATK v4.1.4.1 (McKenna et al. 2010) HaplotypeCaller best practices, by first generating g.vcf files, followed by joint calling of all samples. SNPs and INDELs were extracted and filtered out separately (see Supplementary Materials). Subsequently, we used two filtering strategies to recover high quality biallelic variants, one to characterize standing genetic variation across all environments, and another to identify de novo mutations exclusive to each environment. For the first strategy, we removed sites with missing data using the option *–max-missing* 1 in vcftools v0.1.16 (Danecek et al. 2011), and further filtered out sites where at least one sample had coverage below 25× or above the 95th coverage sample percentile in R, as well as sites that had a mappability of <1 as calculated by GenMap 1.3.0 (Pockrandt et al. 2020), with a resulting median depth of coverage per sample of around 30× to 780× (supplementary fig. S2, Supplementary Material online). From these filtered variant set, we selected sites that showed evidence of being homozygous (MAF < 0.05) in the haploid parental strains SK1 and Y55 (false heterozygous sites would be produced by mismapping and hidden paralogy) and that were also clearly heterozygous in the founder samples (MAF > 0.3), which by design should be exclusively F2 heterozygotes. We observed no obvious large biases in the allele frequencies of the founder populations away from the 0.5 expected mean, which would have otherwise indicated the presence of lethal allelic combinations between SK1 and Y55. The final variant set contained only sites that were shared across all samples in all environments. For the second strategy, we first split the variants by environment using vcftools and remove sites that became monomorphic (based on allele depth, MAF = 0) using the custom script *getvariantspool.py* available in the GitHub repository. We then removed sites with missing data, bad coverage and mappability, and heterozygosity in the parental strains as above. Finally, we used snpEff v5.0e (Cingolani et al. 2012) to annotate the effect of identified variants on genes using the database of the reference genome R64-1-1.99 with canonical transcripts (*-canon*). The workflow is available at https://github.com/SLAment/AdaptationDynamics as Snakemake v. 5.30.1 (Köster and Rahmann 2012) pipelines, which depend on the ggplot2 v. 3.3.53 (Wickham 2016), cowplot v. 1.1.1 (Wilke 2019), dplyr v. 1.0.5 (Wickham et al. 2021), tidyr v. 1.1.4 (Wickham 2021), vcfR v. 1.12.0 (Knaus and Grünwald 2017), and ggpubr v. 0.4.0 (Kassambara 2020) R packages. See Supplementary materials for parameters used.

### Analysis of Standing Genetic Variation

Due to our experimental design, in which only one round of sexual recombination occurred, we expect that large haplotypes were formed in the initial recombination event. To assess the dynamics of these haplotype blocks, we implemented a method of reconstructing selected haplotypes based on correlation between SNPs through time, inspired by Otte and Schlötterer's Haplovalidate (Otte and Schlötterer 2021). We first normalized allele frequency data of the filtered set of SNPs with an arcsine-square-root transformation with subsequent centering and scaling (Otte and Schlötterer 2021). We then calculated correlations between all SNPs and performed hierarchical clustering to obtain haplotype blocks. Only haplotype blocks with at least ten SNPs were retained. We tested various minimum correlation cutoffs for hierarchical clustering from 0.3 to 0.9, and chose the correlation cutoff for each replicate which resulted in the most haplotype blocks of minimum size. We chose one SNP at random within each haplotype cluster to represent the overall cluster trajectory. Note that each haplotype cluster might include multiple chromosomes, as reproduction in our experiment is strictly asexual. This aspect of our data also makes the original HaploValidate algorithm, which relies on linkage equilibrium between chromosomes, non-applicable to our experiment.

The original parental strains, SK1 and Y55, have a digital DNA-DNA hybridization (dDDH) distance of 0.0056. This value was calculated with the Formula 2 (identities/HSP length) of Meier-Kolthoff et al. (2013) on BLAST + alignments in the Genome-to-Genome Distance Calculator (v. 3.0, https://ggdc.dsmz.de/; Meier-Kolthoff et al., 2022). However, preliminary analyses revealed that the divergence between these strains is not uniform along the genome. Instead, the divergence between them is distributed in blocks, with some large chromosomal regions being identical between strains. Thus, our analyses of standing genetic variation below include relatively few sites of some chromosomes (e.g., chromosomes 2, 6, and 10).

In order to explore parallelism in the fixation of standing genetic variation, we used the set of filtered SNPs and divided the genome in non-overlapping 10 kb windows and calculated their median allele frequency. We only retained windows with at least four SNPs (504 out of 1,184 windows or 42.57%). No windows of chromosome 10 were recovered. We classified each window as nearly fixed (MAF ≤ 0.1) for either parental allele or as non-fixed (MAF > 0.1) and then counted how many replicates in each environment fixed for the SK1 allele (added +1 to the count) or to the Y55 allele (−1). Windows that were not fixed for a parental allele were counted as 0. We then scaled this count to range from 1 (all replicates fixed the SK1 allele) to −1 (all replicates fixed the Y55 allele). As a useful comparison, consider that in many replicates a single F2 genotype fixed toward the end of the experiment. For a given locus with two alleles, A and B, the probability of an F2 to be homozygous for one allele (thus appearing as fixed with our index) is (0.5)(0.5) = 0.25, and there are two possible homozygous cases (AA and BB). The probability of two F2s to be homozygous for the same allele would then be equal to (probability of being homozygous in the first F2)(probability of being homozygous in second F2)(probability of being the same allele) = (0.5)(0.5)(0.5) = 0.125. So in total (0.125)(504) = 63 windows should be the same between two different populations where a single diploid genotype dominates.

### Analysis of de novo Mutations

To identify de novo mutations, we compared the alleles of the parental strains SK1 and Y55 with alleles found in the biallelic sites of the evolved samples in the second variant data set. All sites that had an allele not found in any of the parental strains and that had a MAF of <0.3 in the founder populations were considered putative de novo mutations. The MAF requirement excludes a few mutations (29 SNPs and four INDELs) that presumably appeared between the sequencing of the parental strains and the sequencing of the founder populations, which were already at intermediate frequencies (≥0.3) at the beginning of the experiment. Some of these intermediate mutations could be the result of read mismapping and/or paralogs and faulty variant calling. As many of the remaining putative mutations are also either sequencing errors or the result of mismapping as above, we further filtered for mutations that reached a frequency of 10% at least once in the experiment and considered them de novo mutations (310 variants). From this, we identified candidate adaptive mutations by filtering for variants that reached a frequency >35% in at least one sample. The resulting 92 variants were subject to manual curation by examining mapped reads in the Integrative Genome Viewer v. 2.12.2 (Robinson et al. 2017). Sites with reads mapping to multiple locations, variant caller inconsistencies, and sharp increases in coverage were discarded (30 variants). Note that the large proportion of discarded de novo mutations does not reflect the overall quality of the total variant set. Instead, it reflects the fact that bad quality sites have a tendency to create high-frequency variants. Out of the final 62 curated mutations, 57 were located within protein-coding genes. We used the list of these genes as input for the STRING database website (Szklarczyk et al. 2021; https://string-db.org/) to infer functional associations, last consulted on March 7, 2022 with default parameters.

### Analysis of Copy Number Variants and Potential Aneuploidies

To identify potential aneuploidies, we ran samtools depth only counting reads with mapping quality greater than or equal to 20 (-Q 20) for each base pair across the genome. Then each replicate population was analyzed using a custom python pipeline, which determined the mean read depth in 1 kb non-overlapping windows, removed outliers (>2× mean chromosome read depth), determined mean read depth within each chromosome and calculated the mean read depth across the genome for the replicate population. The depth of each chromosome was then compared with the genomic mean depth, and was characterized as a potential aneuploidy in the population if chromosome depth was ±25% of the mean genome depth. For a few sequenced time points, read depth was not uniformly distributed along chromosomes, with chromosome centers having less read depth, resulting in a noticeable “smile” shape in read depth. The cause of the unusual distributions is unknown, but was not linked to overall sample read depth or to sequencing flow cell. For time points where read depth was highly variable within chromosomes (standard deviation >15% of mean read depth and chromosome center >15% lower than chromosome ends), the average chromosome-end read depth (excluding subtelomeric regions) was used as the chromosome mean. These were calculated individually for each replicate/generation time point, and plotted using custom python scripts. We used a similar approach to determine if there were substantial changes in the copy number variation of the ENA gene in any of our populations (Supplementary Materials).

### PCR Verification of Mating Type

Given that changes in allele frequencies of most LiAc0.02 populations suggested haploidy, we tested for the presence of both mating type alleles. We used PCR primers designed to target within the mating loci genes (Illuxley et al. 1990) in generations 30, 60, 100, and 200 from the LiAc 0.02 M environment. Primers were designed to display a band at 492 bp for the MATa or 369 bp for the MATα allele. Two bands on the gel indicate a non-haploid population (likely diploid as in the founder populations). Note that all populations in this experiment were *HO* knock-outs (they cannot switch mating type and self-fertilize), but ploidy changes may still occur. Sporulation and mating with strains of known mating type were also used to test for mating type (see Supplementary Materials for further details).

## Supplementary Material

msac242_Supplementary_Data

## Data Statement

Phenotypic data are available in supplementary table S1, Supplementary Material online. The raw sequencing data were deposited in the European Nucleotide Archive, accession number PRJEB46680. The code used for alignment, variant calling, analyses, and plotting are available at https://github.com/SLAment/AdaptationDynamics.
