# Supplementary material for: The Dynamics of Adaptation to Stress from Standing Genetic Variation and de novo Mutations
Source: Mol Biol Evol. 2022 Nov 5;39(11):msac242. doi: 10.1093/molbev/msac242 (PMC9703598; doi:10.1093/molbev/msac242)
Supplement: msac242_Supplementary_Data [file msac242_supplementary_data.zip › Supplementary_Materials_all_MBE_R1.pdf]

Supplementary Materials for

The dynamics of adaptation to stress from standing genetic variation and *de novo* mutations

S. Lorena Ament-Velásquez, Ciaran Gilchrist, Alexandre Rêgo, Devin P. Bendixsen, Claire Brice, Julie Michelle Grosse-Sommer, Nima Rafati, Rike Stelkens

Fitness Dynamics

Optical density was measured using a microplate reader (Sunrise, Tecan) using the accuracy mode at 600nm absorbance. To do this, frozen population samples were thawed and 10µl was used to inoculate 5ml SC media, then grown for 24h at 30°C with shaking. After 24h, 96-well plates were filled with 148µl per well of the appropriate medium and inoculated with 2µl of the 24h yeast culture. OD_{600} was measured at t_0 and after 24h of growth at 30°C without shaking. Plates were gently vortexed for 30s and shaken in the plate reader for 20s immediately before OD readings. Each replicate population was measured in five wells and normalised using blank wells containing only media. Founder populations were measured separately, with 50 wells per environment in order to capture the potential phenotypic diversity present in the founding population.

DNA extraction and whole genome sequencing

For DNA extraction, 10µl of frozen populations were grown in 5ml SC media at 30°C shaking at 200 rpm for 24-48h (until near saturation) and ~1ml of the culture was used for DNA extraction using Thermo Scientific KingFisher™ Duo Prime Purification. First the culture was centrifuged at 13000rpm for 5 minutes, and the resulting cell pellet incubated in zymolyase solution for 30 minutes at 37°C. After this, cells were then centrifuged again at 13000rpm followed by washing in 1ml of sterile deionised H_2O, and centrifugation. 200µl of cell lysis buffer and 25µl of proteinase K solution (from the manufacturer's kit) was added to the cell pellet and mixed by pipette, followed by thorough vortexing for 5 minutes. These were then incubated at 70°C for 10-15 minutes, with occasional centrifugation. After incubation, samples were vortexed for 30s and 10µl of RNAse solution added for 10 minutes at room temperature. Samples were then centrifuged for 5 minutes at 6000xg. 225µl of the supernatant (lysed sample) was then added to wells in a plate containing 25µl of magnetic beads and 360µl of binding buffer (from the manufacturer's kit). These samples were then extracted using the KingFisher™ Duo Prime's standard DNA extraction programme, including three washes to remove impurities. Finally, samples were eluted into the final tubes containing 100µl of elution buffer (from the manufacturer's kit).

All library preparation and sequencing were performed at the Science for Life Laboratories (Stockholm, Sweden).

Quality control, mapping and variant calling

Trimmomatic v0.36 (Bolger et al., 2014) used the following parameters: ILLUMINACLIP:adapters.fa:2:30:10:8:true SLIDINGWINDOW:4:15 LEADING:30 TRAILING:30 MINLEN:50 HEADCROP:10.

GATK v4.1.4.1 (McKenna et al., 2010) HaplotypeCaller used the following cut-offs to separately extract and filter SNPs and INDELs: SNPs – QD<2.0, FS > 10.0, ReadPosRankSum <-3.0, MQRankSum < -6.0, SOR > 3.0, MQ < 40.0; INDELs – QD <10.0, FS > 10.0, ReadPosRankSum <-4.0, MQRankSum < -8.0, SOR > 4.0, MQ < 40.0.

Preliminary analyses revealed indications of contamination in a few samples. Namely, in some samples the majority of reads mapped to another yeast species also present in the lab environment (*Saccharomyces paradoxus*) or multiple samples shared sets of *de novo* mutations that appeared simultaneously in the last time points of some populations, while at the same time exhibiting a sudden increase in allelic diversity across the genome. We also compared the allele frequencies along the genome for all time points, and found that a few samples were identical. As we deemed these observations unlikely to evolve naturally in multiple populations simultaneously, we excluded two NaCl-adapted populations after 700 generations, all samples at generation 1000 from the LiAc treatments, as well as all time points of one NaCl population (replicate 5) and one LiAc 0.01M population (replicate 3). All analyses were rerun without these samples.

Analysis of de novo mutations

We calculated the probability of mutations affecting the same gene independently as follows (Huang et al., 2018):

(Freq. of mutations per gene in lineage 1)(Freq. of mutations per gene in lineage 2)*Total No. of genes*

We assumed the total number of genes to be 6400 as in the S288C reference genome (Genbank GCF_000146045.2). As an example, consider the gene *CYC8*, which had two independent mutations in replicates 2 and 3 of the LiAc 0.02M environment. As there were five and four high frequency (> 0.35), manually curated mutations in these two replicates at the end of the experiment, respectively, the probability of observing two independent mutations in a gene of these two replicates is: (5/6400) x (4/6400) x (6400) = 0.0031. Note, however, that a total of 6400 genes leads to an underestimation of this number because many sites across the genome will not be callable due to issues with coverage and

mismapping. Still, even using as few as 1000 genes leads to probabilities lower than 0.03 for all genes with multiple hits (not shown).

Analysis of copy number variants and potential aneuploidies

Like for chromosomal aneuploidies, we used a depth of coverage approach to determine if there were substantial changes in the copy number variation of the *ENA* gene in any of our populations. The genome assemblies of SK1 (Yue et al., 2017) and Y55 (Bendixsen et al., 2021) contain a single *ENA* copy (identical between strains), while the reference genome we used for variant calling (S288c) contains three divergent ENA copies, likely introgressed from *S. paradoxus* (Warringer et al., 2011). Thus, we re-mapped all samples to the SK1 genome with BWA and marked duplicates with Picard. We obtained read counts per base as above with samtools for the coordinates chrIV:432983-636260, containing the *ENA* locus, plus 100 kbp to the right and to the left of that gene. We further removed regions with Genmap's mappability lower than 1 (a total of 2094 bp) using BEDtools v. 2.29.2 (Quinlan & Hall, 2010). For every sample, the relative depth of the *ENA* gene was calculated as the mean depth of coverage within the *ENA* locus divided by the mean depth of coverage of the flanking regions, such that a value of 1 indicates no copy number variation. As a comparison, we also calculated the relative depth of the *ENA* gene for the reads of SK1 and Y55, which are haploid and have a single copy, and for S288c (European Nucleotide Archive run SRR2968033) that has three divergent copies.

Verification of mating type by sporulation and mating

To identify diploid *MATa/MAT*α populations, we sporulated populations on KAc agar plates for 72h. We then looked for the presence of tetrads using a microscope. The formation of tetrads in a population confirms the presence of diploid *MATa/MAT*α cells within the population. The absence of tetrads indicates either all cells contain only one mating type (either haploid or homozygous diploid for *MATa* or *MAT*α) or that there are problems with sporulation in the populations.

Populations identified as potentially haploid were then mixed with "tester strains" of a known mating type, and pipetted onto a YPD agar plate. After 24h at 30°C, these were replica plated to a KAc agar plate. Mating type was identified upon successful mating with a "tester strain". For example, if the haploid population successfully mates with a "tester strain" known to be *MATa*, the haploid population will be *MAT*α.

References

Bendixsen, D. P., Gettle, N., Gilchrist, C., Zhang, Z., & Stelkens, R. (2021). Genomic Evidence of an Ancient East Asian Divergence Event in Wild *Saccharomyces cerevisiae*. *Genome Biology and Evolution*, *13*(2). https://doi.org/10.1093/gbe/evab001

Bolger, A. M., Lohse, M., & Usadel, B. (2014). Trimmomatic: A flexible trimmer for Illumina sequence data. *Bioinformatics*, *30*(15), 2114–2120. https://doi.org/10.1093/bioinformatics/btu170

Huang, C. J., Lu, M. Y., Chang, Y. W., & Li, W. H. (2018). Experimental Evolution of Yeast for High-Temperature Tolerance. *Molecular Biology and Evolution*, *35*(8), 1823–1839. https://doi.org/10.1093/molbev/msy077

McKenna, A., Hanna, M., Banks, E., Sivachenko, A., Cibulskis, K., Kernytsky, A., Garimella, K., Altshuler, D., Gabriel, S., Daly, M., & DePristo, M. A. (2010). The genome analysis toolkit: A MapReduce framework for analyzing next-generation DNA sequencing data. *Genome Research*, *20*(9), 1297–1303. https://doi.org/10.1101/gr.107524.110

Quinlan, A. R., & Hall, I. M. (2010). BEDTools: A flexible suite of utilities for comparing genomic features. *Bioinformatics*, *26*(6), 841–842. https://doi.org/10.1093/bioinformatics/btq033

Warringer, J., Zörgö, E., Cubillos, F. A., Zia, A., Gjuvsland, A., Simpson, J. T., Forsmark, A., Durbin, R., Omholt, S. W., Louis, E. J., Liti, G., Moses, A., & Blomberg, A. (2011). Trait Variation in Yeast Is Defined by Population History. *PLoS Genetics*, *7*(6), e1002111. https://doi.org/10.1371/journal.pgen.1002111

Yue, J. X., Li, J., Aigrain, L., Hallin, J., Persson, K., Oliver, K., Bergström, A., Coupland, P., Warringer, J., Lagomarsino, M. C., Fischer, G., Durbin, R., & Liti, G. (2017). Contrasting evolutionary genome dynamics between domesticated and wild yeasts. *Nature Genetics*, *49*(6), 913–924. https://doi.org/10.1038/ng.3847

Supplementary Figures and Tables

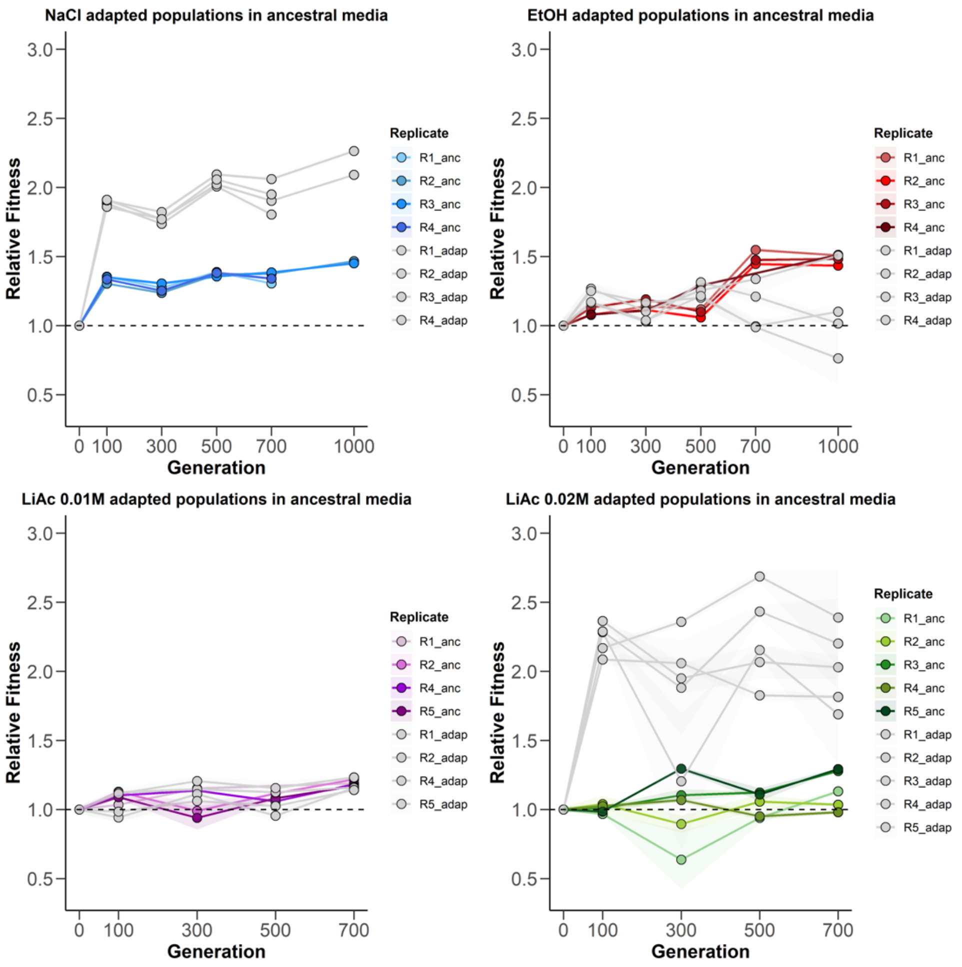

Supplementary Figure 1. Mean relative fitness (optical density after 24h vs. founder populations) of evolved populations in ancestral (SC media) and selective conditions. Evolved populations tested in ancestral conditions are shown in different shades of colour (NaCl = blue, EtOH = red, LiAc 0.01M = purple, LiAc 0.02M = green). Evolved populations tested in selective environments are shown in grey (cf. Figure 2). Shaded areas are 95% confidence intervals. One EtOH population went extinct in the first 100 generations. Two NaCl populations after 700 generations were removed due to contamination.

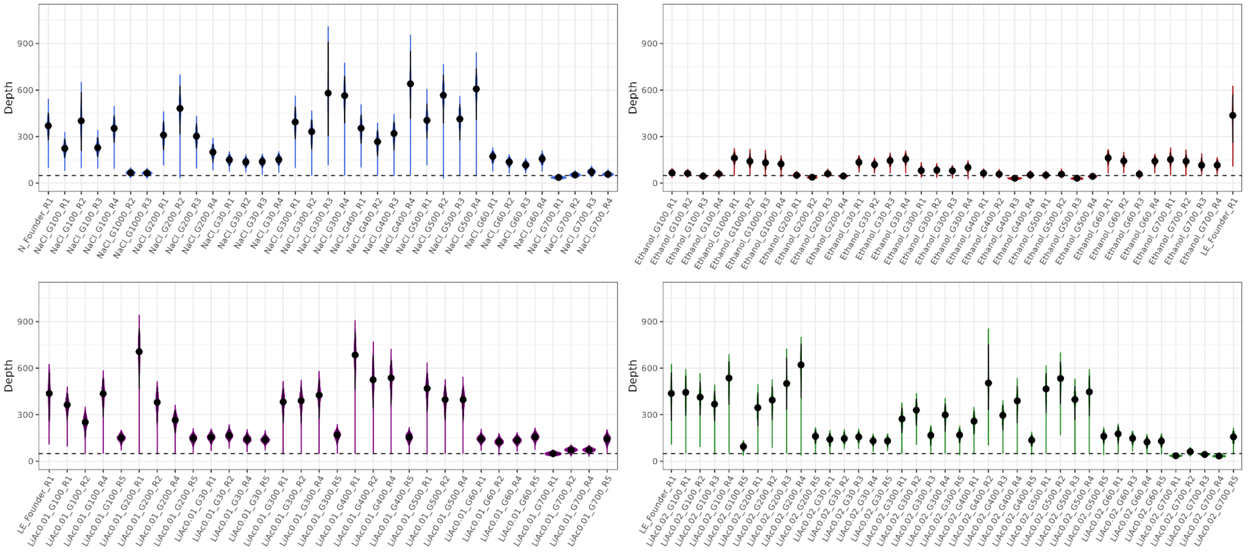

Supplementary Figure 2. Depth of coverage distribution of SNPs per sample. These distributions represent the sets of SNPs after we removed sites with missing data using the option *--max-missing* 1 in vcftools, those that had a depth below 25x or above the 95th coverage sample percentile, as well as sites that had a mappability of less than 1.

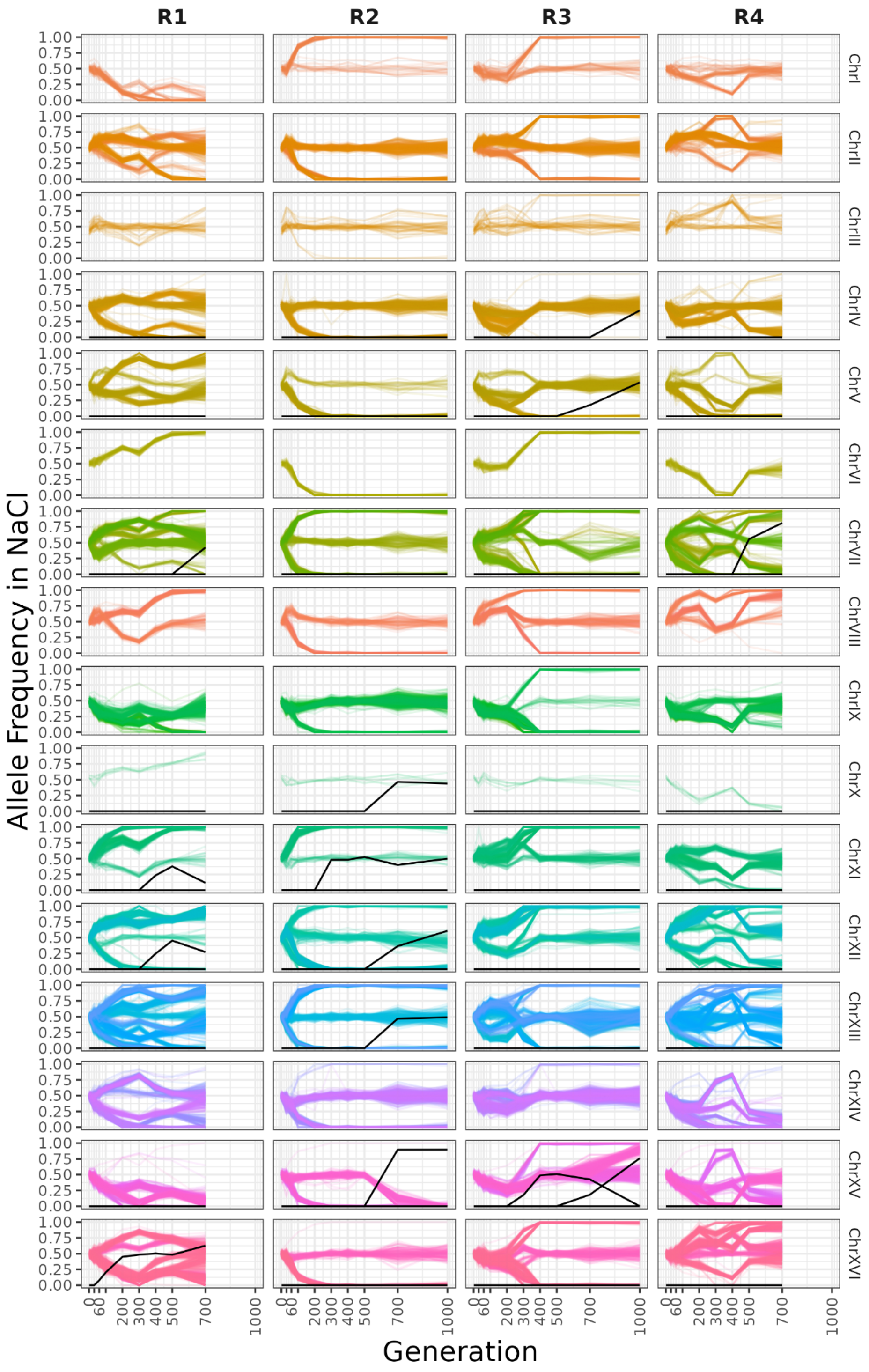

Supplementary Figure 3. Allele frequency trajectories from the ancestral SNP variation (colours) and *de novo* mutations (MAF >0.35, black) in the NaCl environment. Lines connect the allele frequencies of all sites per chromosome and per replicate. Only the allele of the parental strain SK1 is shown in the case of ancestral variation.

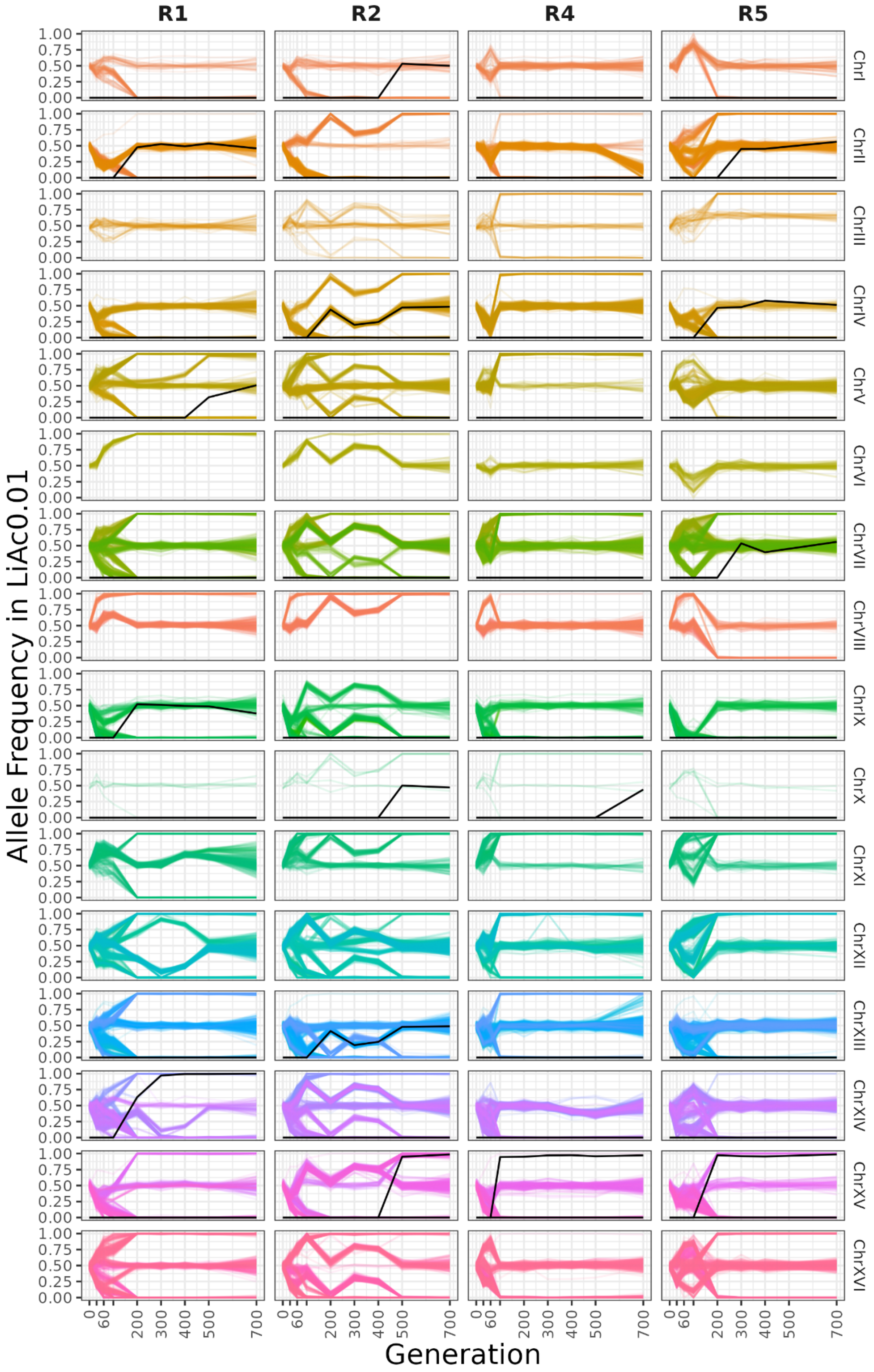

Supplementary Figure 4. Allele frequency trajectories from the ancestral SNP variation (colours) and *de novo* mutations (MAF >0.35, black) in the LiAc 0.01M environment. Lines connect the allele frequencies of all sites per chromosome and per replicate. Only the allele of the parental strain SK1 is shown in the case of ancestral variation.

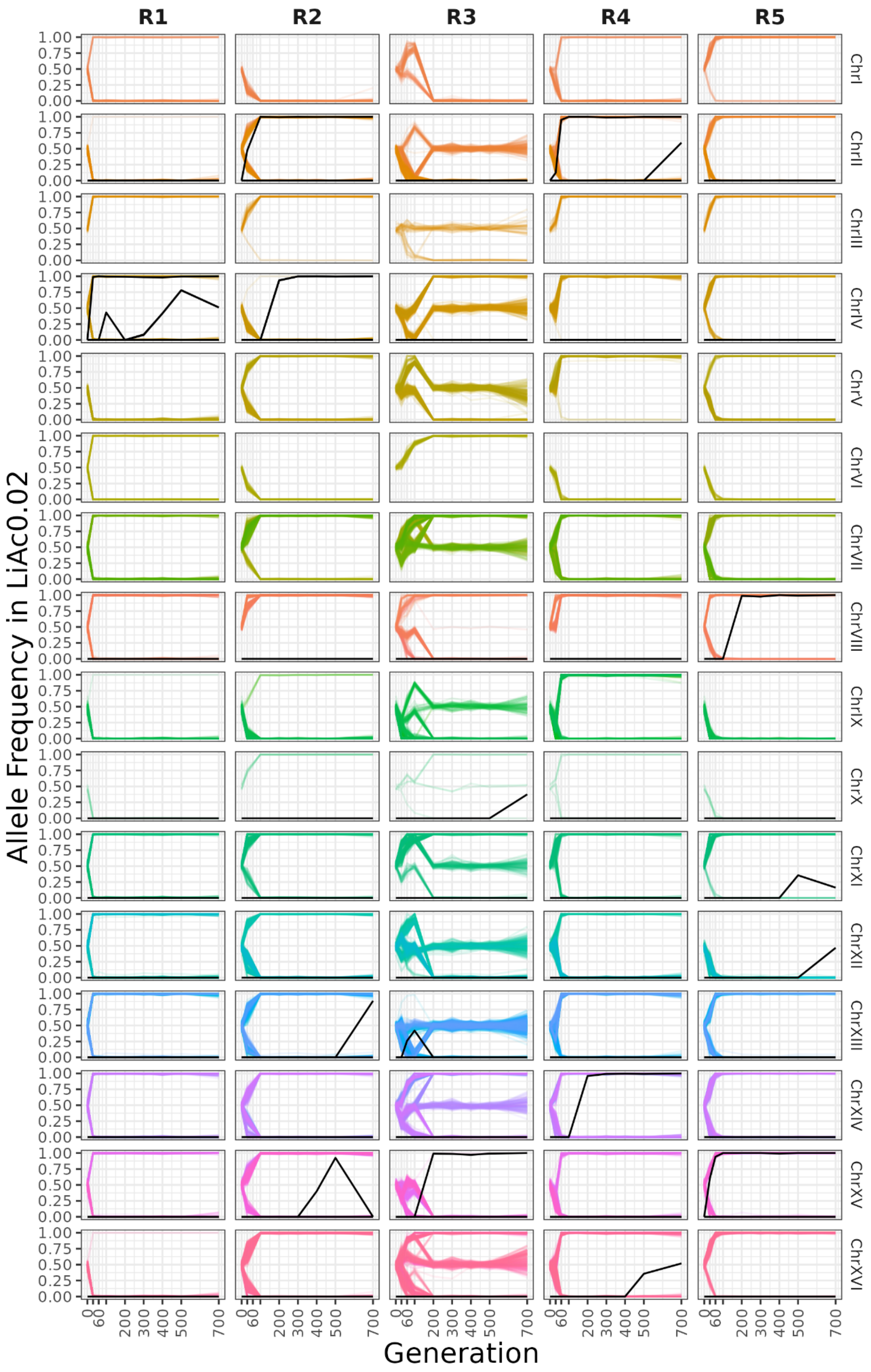

Supplementary Figure 5. Allele frequency trajectories from the ancestral SNP variation (colours) and *de novo* mutations (MAF >0.35, black) in the LiAc 0.02M environment. Lines connect the allele frequencies of all sites per chromosome and per replicate. Only the allele of the parental strain SK1 is shown in the case of ancestral variation.

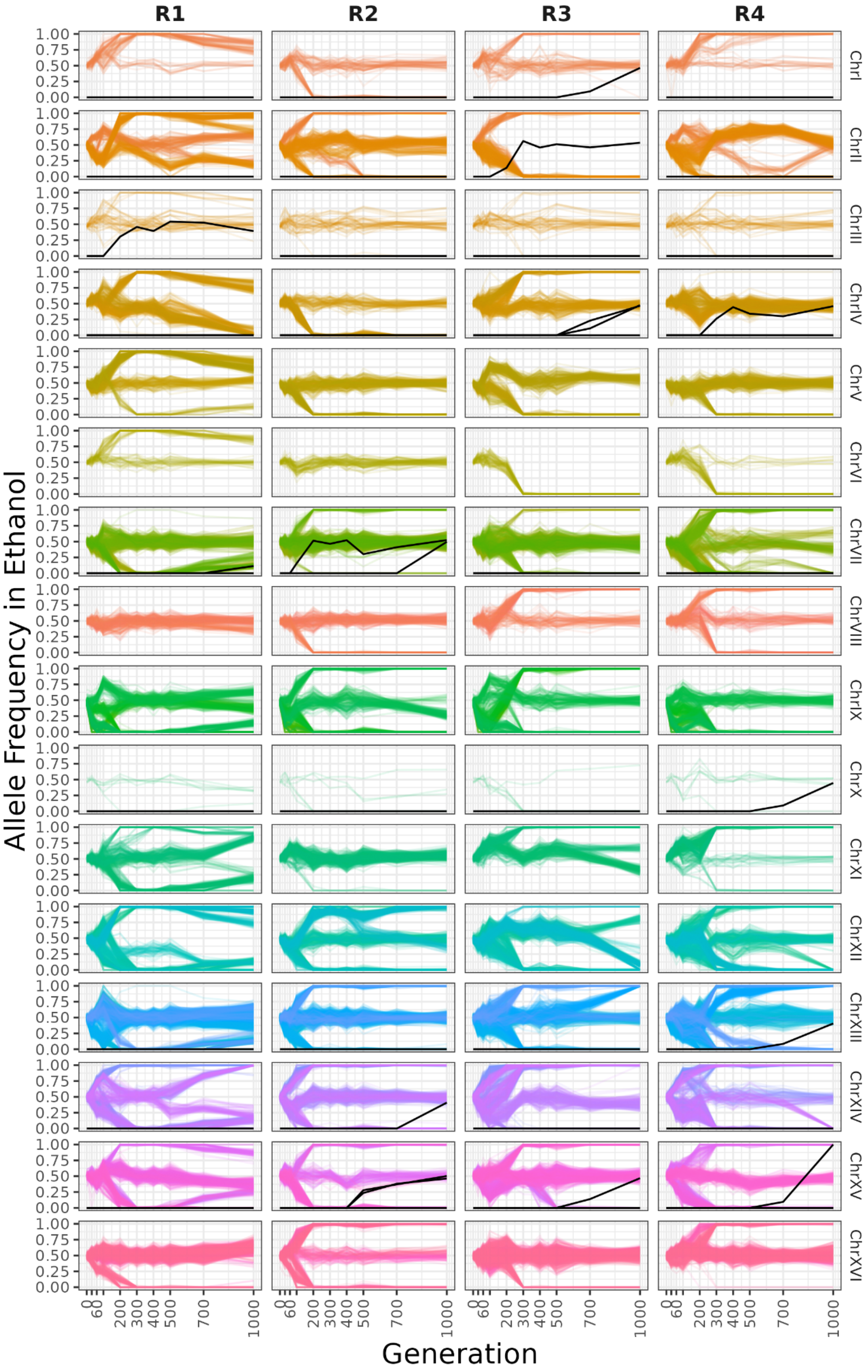

Supplementary Figure 6. Allele frequency trajectories from the ancestral SNP variation (colours) and *de novo* mutations (MAF >0.35, black) in the EtOH environment. Lines connect the allele frequencies of all sites per chromosome and per replicate. Only the allele of the parental strain SK1 is shown in the case of ancestral variation.

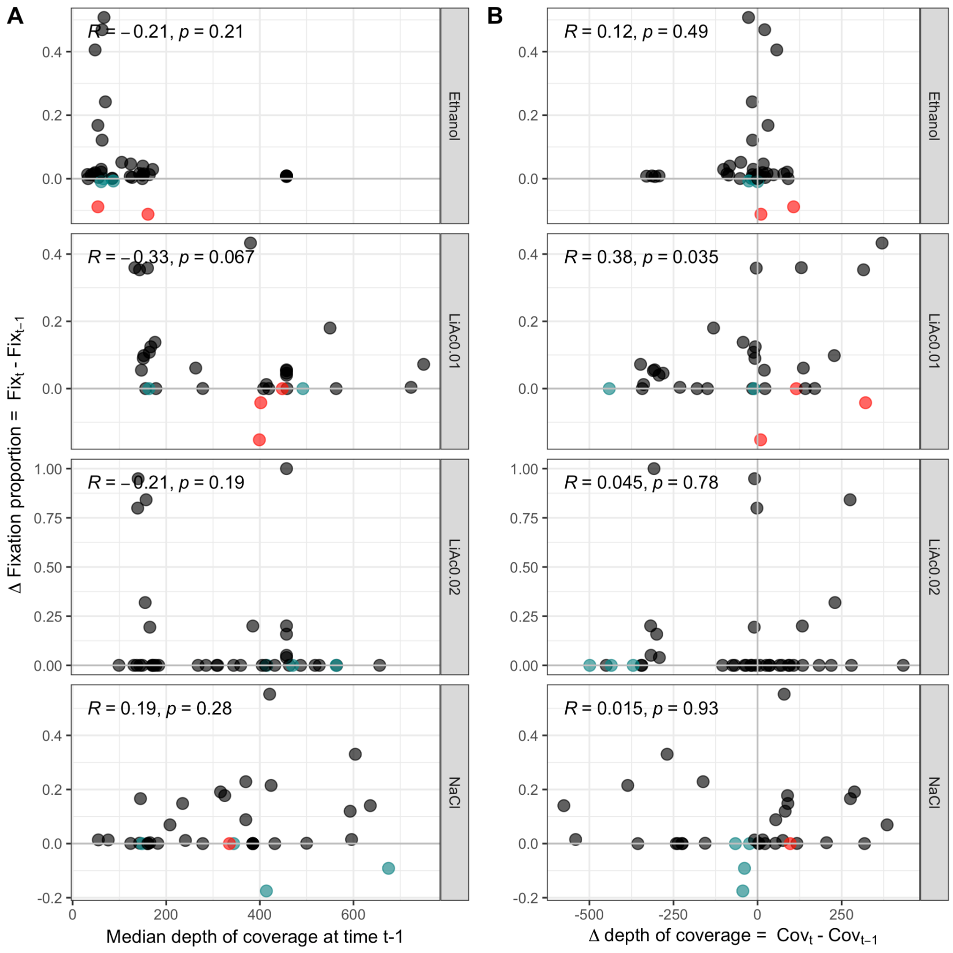

Supplementary Figure 7. Changes in the proportion of nearly fixed (MAF < 0.1) sites is not fully explained by the depth of coverage of a sample. For each sample, we calculated the difference between the proportion of nearly fixed sites at time *t* and the proportion at the previous time *t* - 1 (*ΔFixation proportion*). **(A)** If low coverage is the cause of "unfixing" sites (*ΔFixation proportion* < 0, blue and red points), then we should observe a positive correlation between *ΔFixation proportion* and depth of coverage at *t* - 1, but this does not seem to be the case. Moreover, problematic cases (blue and red points) have a good depth of coverage (>150x) for most treatments, except Ethanol. **(B)** We further calculated the difference in median depth coverage between time *t* and *t* - 1 (*Δdepth of coverage*) and plotted the relationship with *ΔFixation proportion*. Few samples fall into the lower right corner of individual panels (red points), which indicates that negative differences in fixation proportion are not necessarily associated with negative differences in coverage. In fact, the sample with most drastic differences in depth (in LiAc 0.01M) has a relatively small *ΔFixation proportion*. Significance of *p-values* correspond to Pearson correlations.

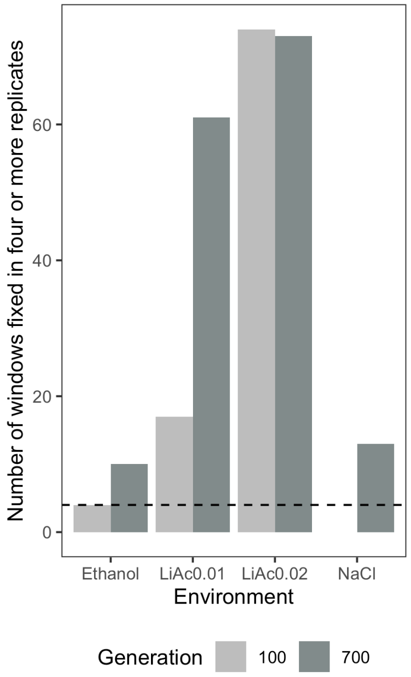

Supplementary Figure 8. Number of non-overlapping 10kb windows that are fixed in four or more replicates for either parental genotype. Notice that all environments had four replicates in total, except for LiAc 0.02M, which had five. As a point of comparison, consider that in most populations a single F2 genotype went to fixation by generation 700. If two populations are compared, what is the probability of two randomly-chosen F2 genotypes sharing windows fixed for a given parental genotype? As their inbreeding coefficient is 0.5, the probability of the two F2s being homozygous for the same allele would then be (Probability of being homozygous in the first F2)(Probability of being homozygous in the second F2)(Probability of being of the same allele) = (0.5)(0.5)(0.5) = 0.125. Using a similar line of thinking, the probability that four independent F2 siblings share the same allele at a given locus is then $(0.5^4)(0.5^3) = 0.0078125$. So we expect 0.78% of the total 504 windows (about 4 windows) to be fixed in four F2 siblings by chance, as marked by the dashed horizontal line.

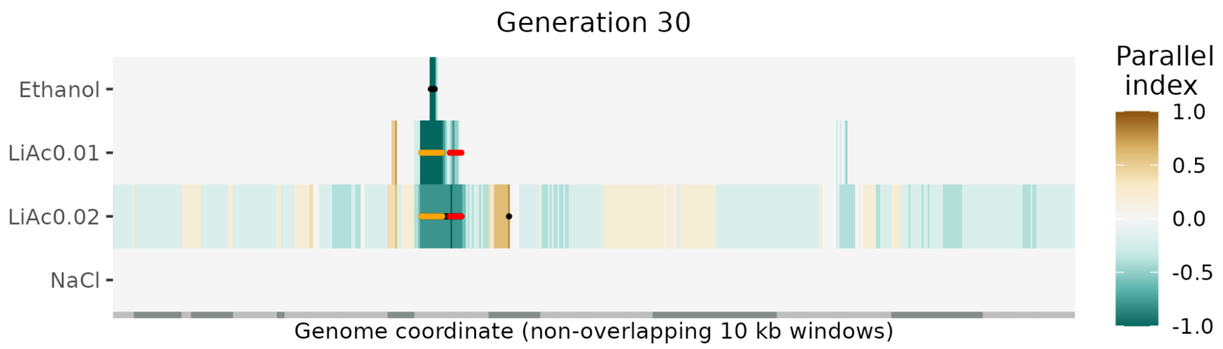

Supplementary Figure 9. Heatmap of genetic parallelism along the genome in the different environments at generation 30. The genome was divided in non-overlapping 10kb windows with at least four SNPs and the median allele frequencies were used to calculate a parallelism index. In essence, the index is a scaled count of how many replicates are fixed (MAF $\leq$ 1) for the SK1 parental allele (+1) or the Y55 parental allele (-1). Chromosome limits are defined by alternating light and dark gray bars at the bottom of the heatmaps. The black points mark windows were either parental allele got fixed in four or more replicates (LiAc 0.02M has five replicates, while the other environments had four). The red points marked windows that were completely fixed in all the LiAc replicates at generation 100. The golden points mark an adjacent region fixed (>4 replicates) in both LiAc environments (and partially EtOH) at generation 30.

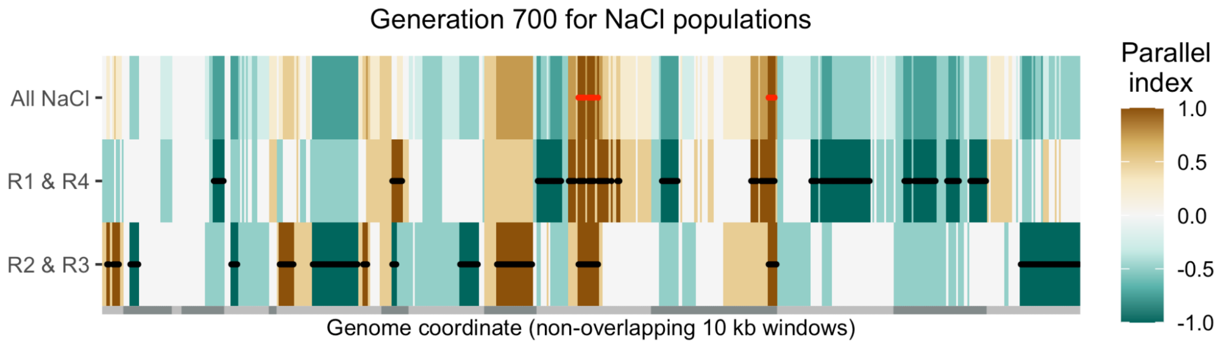

Supplementary Figure 10. Heatmap of genetic parallelism along the genome in the NaCl environments at generation 700. The upper raw contains all four populations, while the middle and lower row compare two sets of populations: one where the two populations are dominated by a single diploid genotype (R2 & R3) and two where more diversity is found (R1 & R4). The genome was divided in non-overlapping 10kb windows with at least four SNPs and the median allele frequencies were used to calculate a parallelism index. In essence, the index is a scaled count of how many replicates are fixed (MAF $\leq$ 1) for the SK1 parental allele (+1) or the Y55 parental allele (-1). Chromosome limits are defined by alternating light and dark gray bars at the bottom of the heatmaps. The black points in the middle and lower rows mark windows were either parental allele got fixed in two replicates. The red points marked windows that were completely fixed in all the NaCl replicates at generation 700.

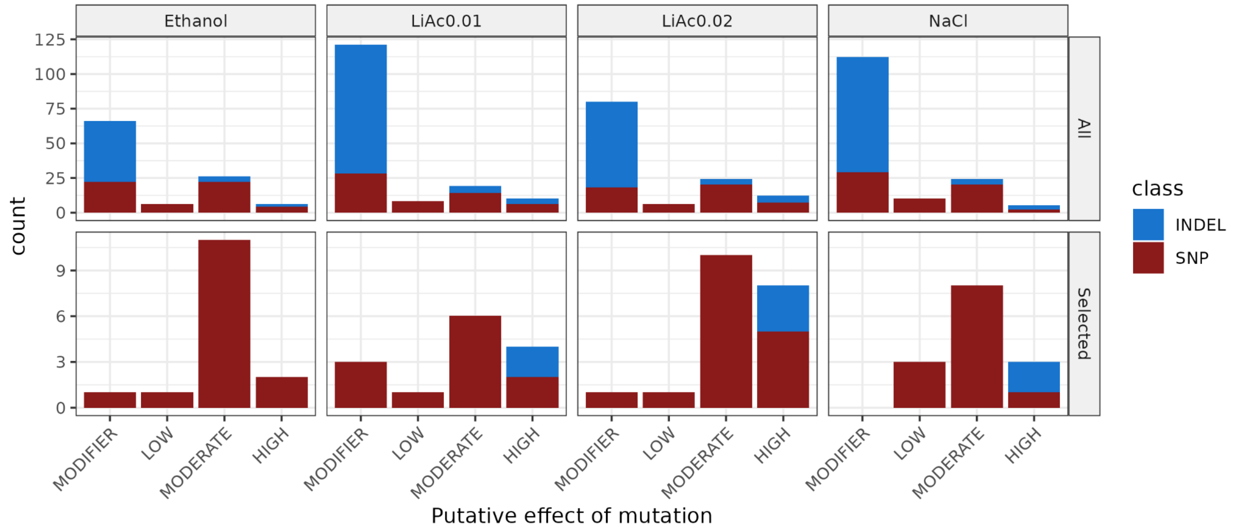

Supplementary Figure 11. Putative fitness effects of de novo mutations as predicted by SnpEff. The upper panel shows the effect of all mutations identified, while the bottom includes only those that reached a frequency of at least 35% during some point of the experiment and that passed the manual curation. “Modifier” indicates mutations falling in the non-coding region of the gene (e.g. enhancers/repressors), “Low” is synonymous mutations, “Moderate” includes missense mutations and in-frame deletions/insertions, and “High” is frameshift variants and gain or loss of stop codons.

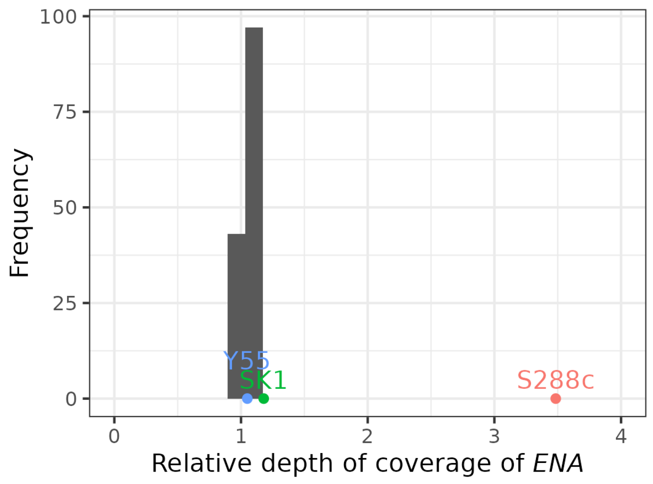

Supplementary Figure 12. Histogram of the relative read depth of the *ENA* gene. For each sample, the ratio between the mean read depth of the *ENA* gene and that of the flanks (about 100 kbp on each side) was calculated. A value near 1 implies no change in the copy number variation. As expected, the haploid ancestral strains SK1 and Y55 present a ratio close to 1, while the strain with three copies, S288c, shows a much higher value.

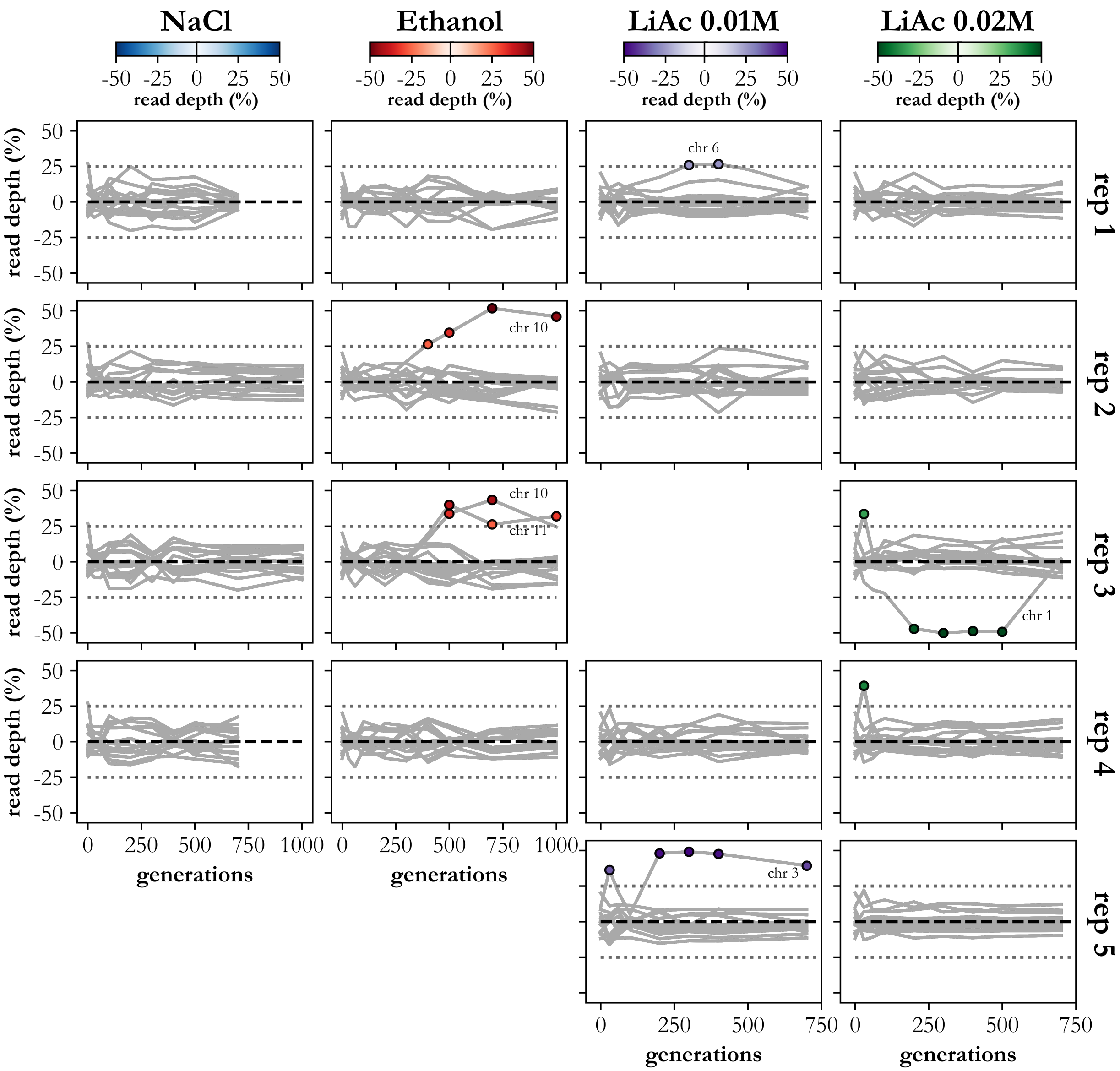

Supplementary Figure 13. Relative chromosomal read depth during adaptation. Lines show the relative read depth of each chromosome (read depth vs. the mean read depth across all chromosomes). Chromosomal deviations greater than 25% from genome read depth are indicated with circles and colour shade indicates the severity of deviation.

Supplementary Table 1. Table of raw yield measurements and relative yield vs. founder, after correction for blank. (Excel file)

Supplementary Table 2. Genes with independent high frequency (>0.35) *de novo* mutations and associated probability of independent mutations per gene.

Gene	Chromosome	No. independent mutations (freq > 0.35)	Lineages where mutations occurred (Total No. mutations)	Multiple mutations probability
CMK2	ChrXV	2	Ethanol_R2 (5) and Ethanol_R3 (5)	0.00390625
CNB1	ChrXI	2	NaCl_R1 (4) and NaCl_R2 (5)	0.003125
CYC8	ChrII	2	LiAc0.02_R2 (5) and LiAc0.02_R4 (4)	0.003125
SNF3	ChrIV	2	LiAc0.02_R1 (4)	0.0025
ISW2	ChrXV	7	LiAc0.01_R2 (5), LiAc0.01_R4 (2), LiAc0.01_R5 (4), LiAc0.02_R3 (3), LiAc0.02_R5 (4), NaCl_R2 (5), NaCl_R3 (4)	1.39698E-19

Supplementary Table 3. Relative read depth calculations for each chromosome. (Excel file)
